# Bionic Ultra‐Sensitive Self‐Powered Electromechanical Sensor for Muscle‐Triggered Communication Application

**DOI:** 10.1002/advs.202101020

**Published:** 2021-06-03

**Authors:** Hong Zhou, Dongxiao Li, Xianming He, Xindan Hui, Hengyu Guo, Chenguo Hu, Xiaojing Mu, Zhong Lin Wang

**Affiliations:** ^1^ Key Laboratory of Optoelectronic Technology & Systems Ministry of Education and International R & D center of Micro‐nano Systems and New Materials Technology Chongqing University Chongqing 400044 P. R. China; ^2^ Department of Applied Physics Chongqing University Chongqing 400044 P. R. China; ^3^ Beijing Institute of Nanoenergy and Nanosystems Chinese Academy of Sciences Beijing 100083 P. R. China; ^4^ School of Material Science and Engineering Georgia Institute of Technology Atlanta GA 30332‐0245 USA

**Keywords:** bionics, human‐machine interfaces, machine learning, Morse code, triboelectric nanogenerators

## Abstract

The past few decades have witnessed the tremendous progress of human–machine interface (HMI) in communication, education, and manufacturing fields. However, due to signal acquisition devices’ limitations, the research on HMI related to communication aid applications for the disabled is progressing slowly. Here, inspired by frogs’ croaking behavior, a bionic triboelectric nanogenerator (TENG)‐based ultra‐sensitive self‐powered electromechanical sensor for muscle‐triggered communication HMI application is developed. The sensor possesses a high sensitivity (54.6 mV mm^−1^), a high‐intensity signal (± 700 mV), and a wide sensing range (0–5 mm). The signal intensity is 206 times higher than that of traditional biopotential electromyography methods. By leveraging machine learning algorithms and Morse code, the safe, accurate (96.3%), and stable communication aid HMI applications are achieved. The authors' bionic TENG‐based electromechanical sensor provides a valuable toolkit for HMI applications of the disabled, and it brings new insights into the interdisciplinary cross‐integration between TENG technology and bionics.

## Graphical abstract

Inspired by frogs’ croaking behavior, a bionic triboelectric nanogenerator‐based ultra‐sensitive self‐powered electromechanical sensor is developed for muscle‐triggered communication human–machine interface (HMI) application. The sensor possesses a high sensitivity, a high‐intensity signal, and a wide sensing range. By leveraging machine learning algorithms and Morse code, the safe, accurate, and stable communication aid HMI applications are achieved.

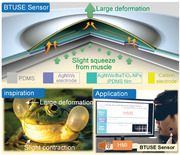

## 1 Introduction

Nowadays, due to the rapid development of science and technology, we humans are experiencing unprecedented convenience. The human‐machine interface (HMI) plays a critical role in providing this convenience by realizing efficient collaboration between humans and the digitalized world.^[^ ^1^, ^2^, ^3^ ^]^ Traditional HMI devices triggered by actions or voices, such as smartwatches, gloves, glasses, and smart robots, are designed for healthy people.^[^ ^4^, ^5^, ^6^, ^7^ ^]^ Patients with amyotrophic lateral sclerosis and those who temporarily or permanently lose their voice due to oropharyngeal cancer treatment cannot enjoy this convenience and even face obstacles in daily communication.^[^ ^8^ ^]^ HMIs based on bioelectrical signals, including neuronal, electroencephalogram,^[^ ^9^ ^]^ electrooculogram (EOG),^[^ ^10^ ^]^ and electromyography (EMG) signals,^[^ ^11^ ^]^ etc., have the potential to address these issues. Among them, the EMG‐based HMI is one of the most effective methods. There are two ways to acquire EMG signals: i) directly record the motor unit action potentials in muscle fibers by using needle electrodes;^[^ ^12^ ^]^ and ii) acquire surface electromyography (sEMG) signals by using surface electrodes. sEMG is widely used in HMI because of its non‐invasive signal acquisition and simple operation.^[^ ^13^ ^]^ However, traditional wet conductive gel silver/silver chloride (Ag/AgCl) electrodes used to obtain sEMG has non‐ignorable disadvantages:^[^ ^14^, ^15^ ^]^ i) the signal strength is very weak and accompanied by noisy signals, which makes it difficult to detect without expensive signal processors; ii) the measurement is unstable, and due to the movement of the muscles and the drying of the gel, the electrode pads may fall off the skin, resulting in a decrease in measurement accuracy; iii) multiple electrodes are required because the recording of EMG shows the potential difference between the two individual electrodes; iv) the mounting locations are not suitable for areas with sharp skin curvatures, such as the face and jaws. Although comfort and measurement accuracy are improved by using ultra‐thin dry electrodes instead of wet electrodes,^[^ ^16^, ^17^ ^]^ the problems of low signal‐to‐noise ratio have not been completely resolved. In addition, the unintentional sEMG output caused by the artifacts of muscle movement may trigger improper operation. Therefore, developing sensitive and accurate sensors for the specific populations discussed above to implement stable and comfortable HMI applications is still challenging.

A triboelectric nanogenerator (TENG) is a new type of energy harvesting device that converts external mechanical energy into electricity by a conjunction of triboelectric effect and electrostatic induction.^[^ ^18^, ^19^, ^20^, ^21^ ^]^ Since TENG was first demonstrated by Professor Zhong Lin Wang in 2012, it has attracted worldwide attention and has been widely used in energy harvesting, wearable electronics, and self‐powered sensing.^[^ ^22^, ^23^, ^24^, ^25^ ^]^ Due to the superior advantages in high‐output performance, stretchable compatibility, and excellent stability, TENG has the potential to face the challenge as an alternative to traditional sEMG and realizes stable and comfortable HMI applications.^[^ ^26^, ^27^, ^28^, ^29^ ^]^ A practical method is to develop TENG‐based micromotion electromechanical sensors.^[^ ^30^, ^31^, ^32^ ^]^ For instance, Professor Hu's group proposed a TENG‐based micro‐motion sensor to replace EOG and convert the eyes’ blink into a high‐performance output control command by fixing it on a pair of hard glasses.^[^ ^32^ ^]^ These early studies proved the potential of TENG‐based sensing technology in detecting the micro‐motion of human muscles. However, due to the buffering of fattiness, muscle fluctuations are usually small. Therefore, in these studies, additional fixation brackets are necessary for the sensor to detect the small muscle fluctuation, which prompted the search for new solutions. Bionics is an important subject in the development of science and technology.^[^ ^33^, ^34^, ^35^ ^]^ Various bionic devices have emerged in an endless stream with unexpectedly excellent performance in recent years, providing technical solutions to many scientific problems.^[^ ^36^, ^37^, ^38^, ^39^ ^]^ These inspired us that the combination of bionics and TENG technology could address the issues of sensing small muscle fluctuations, replacing traditional sEMG devices, and achieving a breakthrough in implementing stable and comfortable HMI applications.

**Figure 1:**
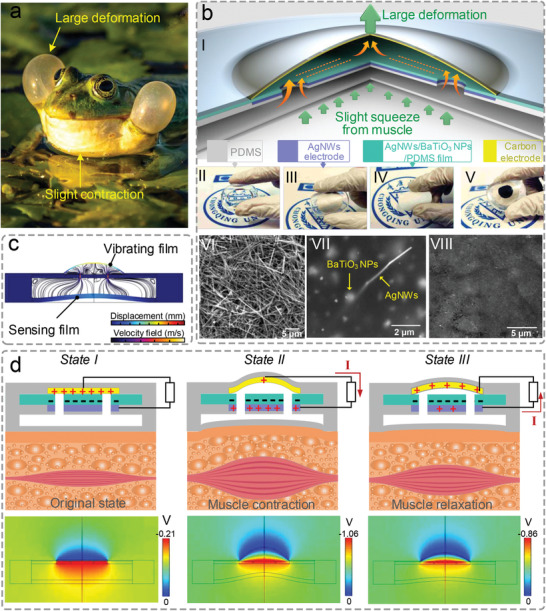
Bionic principle, structure, and working mechanism of the BTUSE sensor. a) Schematic diagram of the croaking behavior of frogs, where slight contraction of the mouth muscles causes large deformation of the external vocal sac. b) Schematic diagram of the proposed BTUSE sensor. I) Schematic structure of the BTUSE sensor, where a slight squeeze from muscles results in asignificant motion of the carbon‐based electrode and then generates high‐intensity electric signals. Optical photos of II) the sensing film, III) bottom electrode, IV) triboelectric layer, and V) the final assembled device. SEM images of VI) AgNWs, VII) AgNWs/BaTiO_3_ NPs/PDMS and VIII) carbon‐based electrically conductive film. c) Velocity field in air and displacement of the vibrating film and the sensing film showing the amplification effect in the BTUSE sensor. d) The principle for the BTUSE sensor generating electrical output signals. Top: Charge behavior of the BTUSE sensor when the muscle is in different states during contraction and relaxation. Bottom: The potential distribution of the corresponding stage calculated by COMSOL.

Here, inspired by the frogs’ croaking behavior, we report a bionic TENG‐based ultra‐sensitive self‐powered electromechanical (BTUSE) sensor for translating the real‐time micromotion of masseter muscle into control command of HMI. When a male frog croaks (**Figure** **1**a), the wide mouth controlled by the muscles contract slightly (micromotion), and the squeezed out airflow pushes the frog's external vocal sac to undergo a significant deformation. Then, the sound of the vocal cords is enhanced by resonating with the periodic deformation. By mimicking the mouth's structure and the vocal sac, a sensing film and a deformable vibrating film are manufactured by flexible PDMS elastomer to amplify the masseter muscle's small fluctuations into the significant motion of vibrating film. TENG technology is ingeniously integrated into the bionic structure to converts the film's vibration into electric signals. The signal intensity is further enhanced by using material modification techniques to maximize the surface charge density of the triboelectric layer. As a result, the output signal intensity of the sensor is 206 times higher than that of the sEMG method. Based on the high‐performance of the BTUSE sensor, we introduce the Morse code as a communication protocol to HMI applications, and successfully develop an authentication system and a hands‐free typing communication system suitable for the disabled. Further, machine learning algorithms are applied to improve user experience and communication efficiency. This work mainly focuses on the detailed study of a bionic sensor from the perspective of the new sensing mechanism and the HMI application. We believe the findings not only provide an alternative to the traditional sEMG technique but also bring new insights into the interdisciplinary cross‐integration between TENG technology and bionics.

## 2 Results and Discussions

### 2.1 Structural Design and Working Principles

In nature, frogs’ croaking behavior is mainly achieved through the cooperation of the mouth and the external vocal sac (Figure 1a). When a male frog croaks, the wide mouth contracts slightly (small motion) due to the muscle contraction, and the squeezed out airflow enters the frog's external vocal sac. As a result, the vocal sac deforms under the push of the airflow, and two huge bubbles (significant deformation) finally appear due to the excellent elasticity and thin thickness. When the deformation frequency matches the vocal cord vibration frequency, the vocal cord's weak sound is greatly amplified and heard.

The BTUSE sensor inspired by frogs’ croaking behavior, as illustrated in Figure 1b, integrates a motion module with an amplification effect and a TENG module that generates electric signals. The motion module comprises a sensing film and a vibrating film separated by a spacer layer, and pores penetrating the spacer layer serve as air‐breathing channels (Figure 1b‐I). Like the frog's mouth, the sensing film with a radius of 14 mm and a distance of 5 mm from the spacer layer moves with muscles’ contraction and relaxation. Similarly, imitating the function of the frog's external vocal sac, the vibrating film with a radius of 7 mm and in close contact with the spacer layer will be greatly deformed under the drive of the sensing film. The shape of all films is chosen to be circular because the circular boundary's curvature is the same compared to other shapes, which results in a small and uniform stress distribution (Note S1, Supporting Information). The TENG module is composed of a silver nanowires (AgNWs)‐based bottom electrode, a silver nanowires/barium titanate nanoparticles/polydimethylsiloxane (AgNWs/BaTiO_3_ NPs/PDMS) composite triboelectric layer, and a carbon‐based top electrode. The bottom electrode and the triboelectric layer are integrated to act as a spacer layer. The top electrode is attached to the vibrating film, and it contacts/separates with the triboelectric material as the film vibrates. The photos in Figure 1b‐II–V clearly show the sensing film, the bottom electrode, the triboelectric layer, and the final assembled device, respectively. The flexibility revealed in the photos is beneficial to advance the user experience of the device. Scanning electron microscope (SEM) images in Figure 1b‐VI–VIII exhibit the surface topography of AgNWs, AgNWs/BaTiO_3_ NPs/PDMS composite triboelectric layer, and carbon‐based electrically conductive film, respectively. Their microscopic morphology affects the signal strength of the device, which will be discussed in detail in the next section.

The amplification effect in the BTUSE sensor stems from the dimension difference between the sensing film and the vibrating film (Figure 1c, Note S2, Supporting Information). Since the air compression in the sensor caused by muscle movement is negligible, the volume of air squeezed out by the slight deformation of the sensing film is equal to that of the vibrating film. It indicates that the small‐radius vibrating film undergoes large deformation when changing the same volume compared with the large‐radius sensing film. The deformation of the vibrating film rises on the increasing dimension difference between the sensing film and the vibrating film (Note S3, Supporting Information). This conclusion is demonstrated by using COMSOL Multiphysics software simulation (Video S1, Supporting Information).

The principle for the BTUSE sensor generating electrical output signals is based on the coupling of contact electrification and electrostatic induction. Figure 1d shows the electricity generation process during muscle contraction and relaxation, including the charge transfer behavior (top) and the potential distribution by COMSOL simulation (bottom). In the initial stage (Figure 1d‐I), there are negative charges on AgNWs/BaTiO_3_ NPs/PDMS film and positive charges on the carbon‐based electrode, which is obtained through several contact–separation cycles between them. In the muscle contraction stage (Figure 1d‐II), the top electrode driven by the vibrating film gradually moves away from the AgNWs/BaTiO_3_ NPs/PDMS layer, resulting in a gradual increase in the potential difference between the two layers. Then, the electrons flow from the top electrode to the AgNWs bottom electrode. In the middle stage of muscular relaxation (Figure 1d‐III), the top electrode gradually returns to the initial state under the sensing film's elastic force and the vibrating film, leading to a gradual decrease in the potential difference. As a result, an opposite current flowing occurs in the external circuit. This is the entire cycle of the signal generation process.

### 2.2 Material Modification of Triboelectric Layers

**Figure 2:**
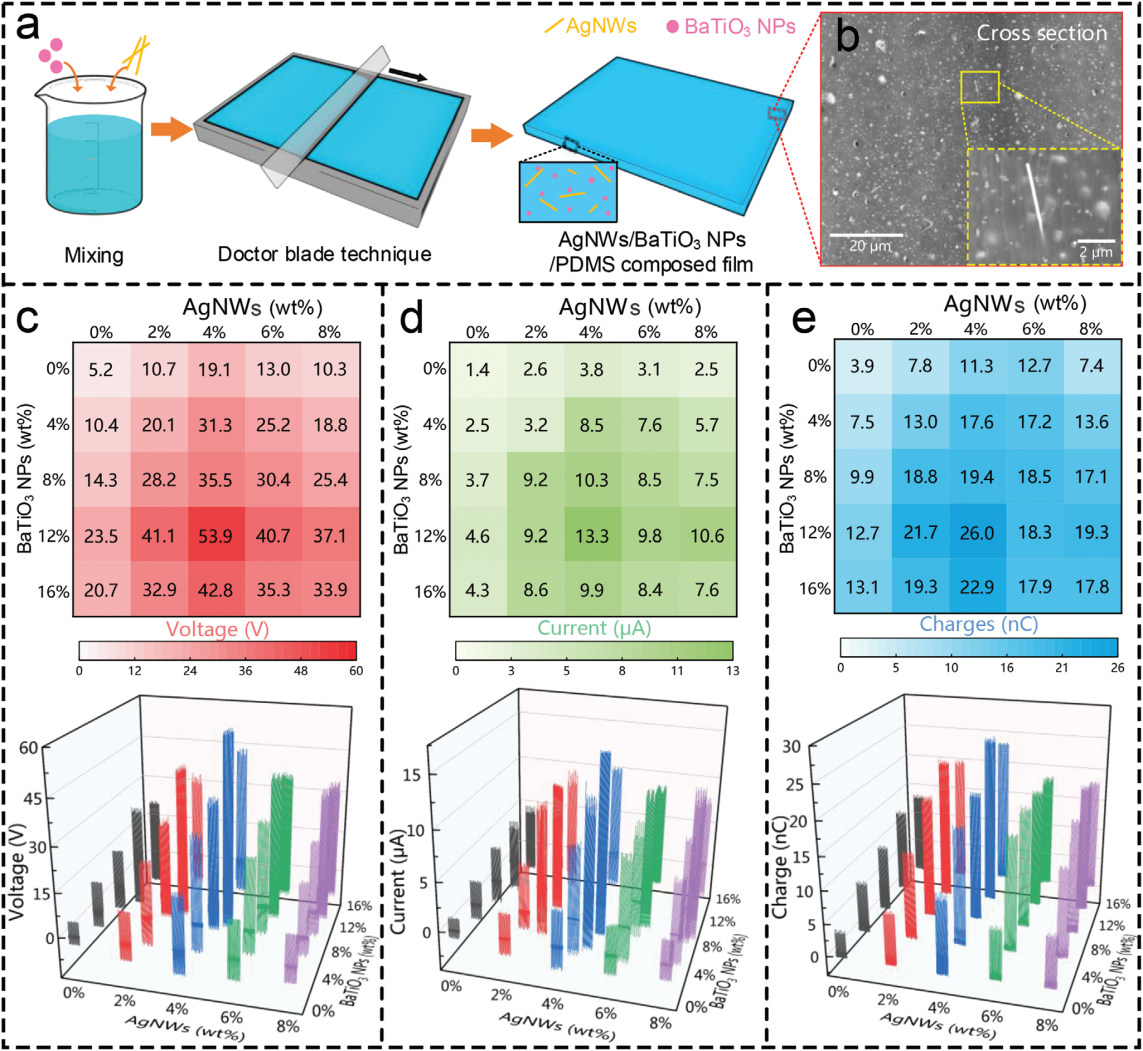
Preparation and characterization of high‐performance dielectric triboelectric materials. a) Schematic diagram of the fabrication process for the AgNWs/BaTiO_3_ NPs/PDMS composite film. b) SEM image showing the details of the cross‐sectional surface topography of the composite film. c) Open‐circuit voltage output of AgNWs/BaTiO_3_ NPs/PDMS composite film with various weight percentages (wt%) of the doped material. The weight percentage of BaTiO_3_ NPs ranges from 0% to 16%, and AgNWs from 0% to 8%. d) Short‐circuit current and e) transferred charge quantity of the composite film.

The output of the BTUSE sensor is closely related to the surface charge density of the dielectric triboelectric material layer. To improve the electrical output performance, we enhance the surface charge density of the triboelectric material layer by using material modification techniques. Specifically, BaTiO_3_ NPs with a high dielectric constant (*ε*_r_ = 150) and AgNWs with high conductivity are chosen as dopants to change the triboelectric material layer's dielectric properties, thereby increasing the surface charge density of the triboelectric film. The preparation of AgNWs/BaTiO_3_ NPs /PDMS composite film includes two steps (**Figure** **2**a): i) filling BaTiO_3_ NPs (AgNWs) into the PDMS matrix; and ii) scraping and then curing the composite film at 55 °C (see Experimental Section for details). The cross‐sectional SEM image of the prepared composite film is shown in Figure 2b, and the weight percentages of BaTiO_3_ NPs, AgNWs, and PDMS in the composite film are 12%, 4%, and 84%, respectively. It can be seen that BaTiO_3_ NPs and AgNWs are uniformly dispersed into the PDMS matrix, indicating the effectiveness of the proposed preparation method. The SEM morphological characterization and X‐ray diffraction (XRD) spectrum of the doped material (BaTiO_3_ NPs and AgNWs) are shown in Note S4, Supporting Information. For the purpose of better identifying the distribution of each component, elemental mapping analysis of the cross‐section of the AgNWs/BaTiO_3_ NPs/PDMS composite film is shown in Note S5, Supporting Information. Clearly, BaTiO_3_ NPs and AgNWs are dispersed into the PDMS matrix and interwoven with each other.

To investigate the influence of the doped materials’ weight percentages (wt%) on the composite films’ surface charge density, we have systematically tested the electrical output of AgNWs/BaTiO_3_ NPs /PDMS composite films with different doping ratios. To eliminate interference from other variables, the experimental platform and test environment used to measure the electrical output were kept unchanged (see Experimental Section for details). The measured results of all the composite films, including voltage, current, and charge, are shown in Note S6, Supporting Information. For the sake of observation, the average value of the electrical output peak within 1s is extracted and plotted into a 2D color map (Figure 2c–e). Then, take the voltage as an example to analyze the influence of the weight percentage of the doped material on the composite film (Figure 2c). In BaTiO_3_ NPs /PDMS binary composite films (0% AgNWs), the output voltage increases first and then decreases with the increase of the weight percentage of BaTiO_3_ NPs, and reaches the maximum when the weight percentage of BaTiO_3_ NPs is 12%. In AgNWs/PDMS binary composite films (0% BaTiO_3_ NPs), the output voltage undergoes the same evolution as the weight percentage of AgNWs increases and reaches the maximum when the weight percentage of AgNWs is 4%. When BaTiO_3_ NPs and AgNWs are simultaneously doped into the PDMS matrix, the output voltage is almost equal to the sum of the outputs when BaTiO_3_ NPs and AgNWs are doped alone. When the weight percentage of BaTiO_3_ NPs and AgNWs are 12% and 4%, respectively, the output voltage reaches the maximum value of 53.9 V, which is 10.36 times higher than that of pure PDMS film. Similarly, the output current and charge undergo the same evolution process as the voltage when the weight percentage of BaTiO_3_ NPs and AgNWs changes (Figure 2d,e).

Next, the output enhancement mechanism of PDMS by using material modification techniques is investigated. TENG's electrical output performance is determined by the surface charge density of the triboelectric material. The maximum surface charge density is proportional to *ε*/*d*, where *ε* and *d* are the relative dielectric constant and effective dielectric thickness of the triboelectric film. Therefore, we can improve the surface charge density by increasing *ε* or reducing *d*, thereby enhancing the output performance of the device. In this work, the *ε* is increased by doping BaTiO_3_ NPs with a high dielectric constant (*ε*_BaTiO3_ = 150 >> *ε*_PDMS_ = 3), and the *d* is reduced by doping AgNWs. The simultaneous doping of BaTiO_3_ NPs and AgNWs into PDMS leads to an increase in *ε* and a decrease in *d*. As a result, the output performance of the triboelectric film is greatly improved through their synergistic effect, which is consistent with the measured results in Figure 2.

### 2.3 Device Optimization and Characterization

**Figure 3:**
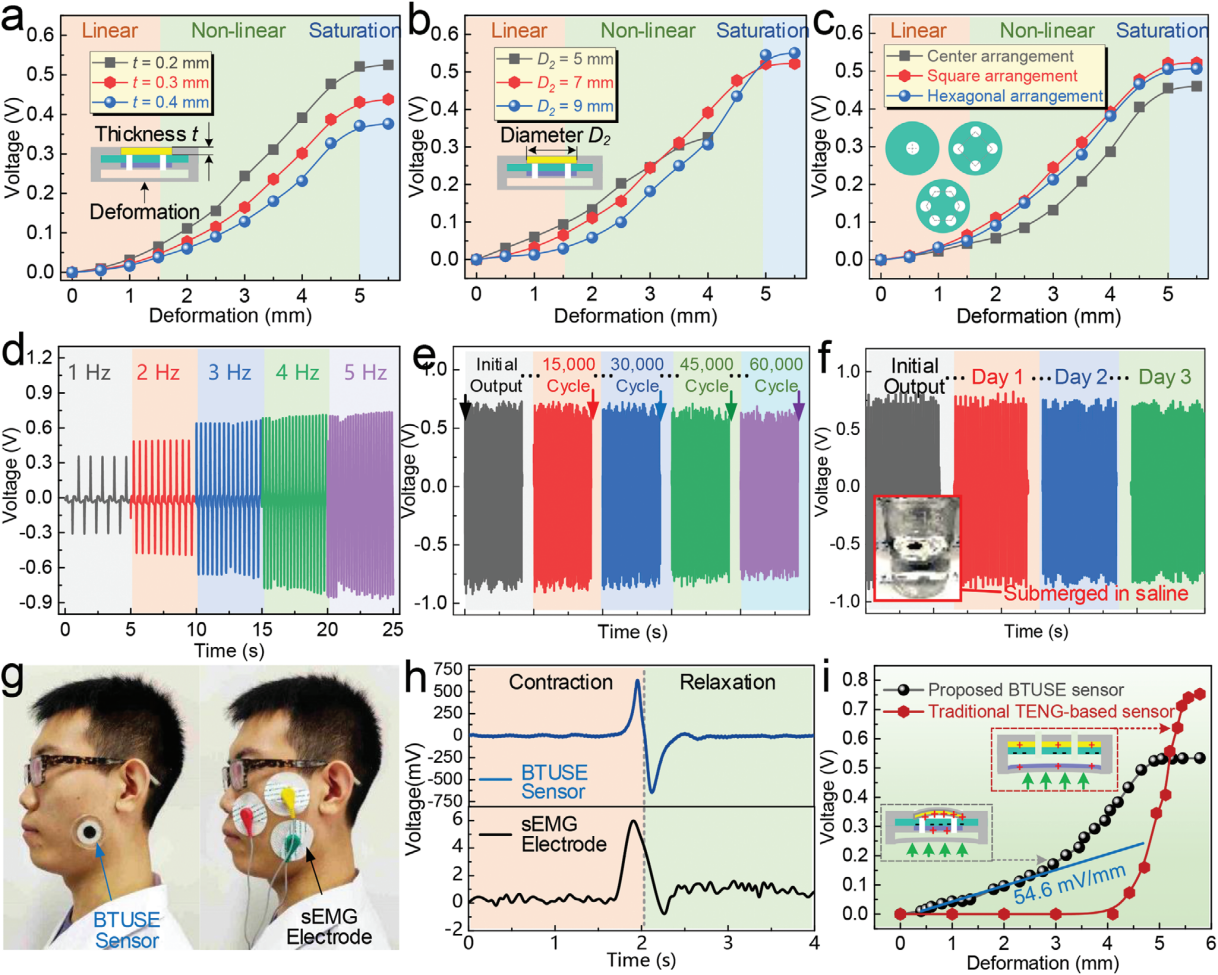
Characterizing the performance of the BTUSE sensor. The influence of a) the film thickness, b) the film diameter, and c) the arrangement of breathing holes on the performance. *t*: the thickness of the vibrating film. d) Open‐circuit voltage of the BTUSE sensor under different deformation frequencies from 1 to 5 Hz. *D_2_*: the diameter of the vibrating film. e) Open‐circuit voltage of the BTUSE sensor that lasted 60 000 working cycles. f) Open‐circuit voltage of the BTUSE sensor that was fully submerged in sweat (normal saline) for 1 day, 2 days, and 3 days. g) Demonstration of collecting masseter muscle motion signal through the BTUSE sensor or the sEMG electrode. h) Corresponding voltage signals from BTUSE sensor or sEMG electrode. i) The performance comparison between the BTUSE sensor and the traditional TENG‐based sensor.

In addition to the influence of the triboelectric material on the output of the BTUSE sensor, it is also critical to investigate the impact of the device structure size on the output, including the dimension and thickness of the vibrating film, and the arrangement of breathing holes. To quantitatively characterize the performance of the sensor, a vibration platform and a displacement sensor are used to control the deformation of the vibrating film (see Experimental Section for details). In the experiment, a common phenomenon was discovered: as the deformation increases, the output of sensors with different configurations undergoes three stages of evolution, namely linear increase, nonlinear increase, and saturation. These three stages correspond to the BTUSE sensor working in small, large, and excessive deformation driven by the muscle, respectively. As verification, the calculated result shown in Note S7, Supporting Information, matches well the experimental results. **Figure** **3**a shows the influence of the thickness of the vibrating film (0.2, 0.3, and 0.4 mm) on the output signal of the BTUSE sensor. As observed, higher sensitivity and signal intensity are obtained with a thinner film configuration. This is because the thinner film deforms more severely under the same driving force, thereby making contact‐separation with triboelectric material more sufficient. It is also noted that the film is prone to cracking when deformed drastically with an excessively thin thickness (less than 0.2 mm). Therefore, 0.2 mm is considered as the optimal thickness. Figure 3b describes the influence of the area of the vibrating film (radius: 5, 7, and 9 mm) on the output signal while keeping other dimensions unchanged. In the stage of linear output, a smaller area means higher sensitivity, since a large deformation is obtained when the area difference between vibrating film and sensing film is large. In the stage of nonlinear increase and saturation, a larger area means higher sensitivity, because a larger area accumulates more charge when the deformation is sufficient. Noted worthily, the sensor with a film radius of 5 mm is prone to cracking after deforming more than 4 mm. Therefore, 7 mm was chosen as the final radius dimension. Figure 3c illustrates the effect of breathing holes in different arrangements (central, square, and hexagonal arrangement) on the output signal. In the linear output stage, the effect of the arrangement on the output signal is negligible, while in the nonlinear or saturation stage, the square arrangement has the best output. It can be understood as the gas in the device flows smoothly when the deformation is small, resulting in little output difference, while when the deformation is large, the film movement of the sensor with fewer breathing channels is partially hindered. However, the channel's increase will reduce the area of the triboelectric material, resulting in a weaker output signal. As verification, the calculated result shown in Note S8, Supporting Information, matches well with the above experimental results. According to the results in Figure 3, the BTUSE sensor with a film thickness of 0.2 mm, a radius of 7 mm, and a square arrangement achieves high output and excellent sensitivity.

After the device optimization, we further investigate its characterization, including frequency response, stability, and waterproofing. As observed, the output of the BTUSE sensor increases as the frequency rises before 4 Hz (Figure 3d, Note S9, Supporting Information). To study the stability and durability in long‐term practical applications, a repeated deformation experiment of 60 000 cycles is carried out at a frequency of 5 Hz (Figure 3e). Clearly, the output maintains stable throughout the whole process. The small attenuation near 60 000 cycles is caused by the irreversible deformation of the sensor. Since the outer layer of the BTUSE sensor is a waterproof PDMS, the output performance of the sensor does not decrease after being immersed in water (Figure 3f), which indicates that the sensor can work stably in a sweaty environment. According to the experiment on temperature and atmospheric humidity, the output voltage increases slightly as the temperature rises within the usual temperature range. No significant changes in the output voltage are observed when the humidity changes within the normal humidity range (Note S10, Supporting Information). Additionally, synchronous measurements are performed to compare the output of the BTUSE sensor and the conventional sEMG device. Figure 3g illustrates the placement of BTUSE sensors and traditional sEMG electrodes. As observed, the proposed BTUSE sensor is more friendly, comfortable, and beautiful to use. Comparing their output signals (Figure 3h), the output signal strength of the BTUSE sensor is 206 times higher than that of the sEMG device. The ultra‐high output can greatly simplify the signal processing circuit and thereby reduce the detection cost. In addition, compared with the traditional TENG‐based two‐electrode sensor (Figure 3i, Video S1 and Note S11, Supporting Information), the BTUSE sensor exhibits a wider detection range (0–5 mm). Especially in the small deformation stage, the BTUSE sensor still maintains a considerable output intensity due to the amplification effect of the ingenious bionic structure. The sensitivity of the BTUSE sensor at the linear stage is calculated to be 54.6 mV mm^−1^. However, the traditional TENG‐based sensor generates an output signal in a narrow range only when the electrode is close to the triboelectric material. Because the long distance between the electrode and the triboelectric material causes weak electrostatic induction and negligible contact electrification, leading to no signal output. On the contrary, the BTUSE sensor always has a contact‐separation action between the electrode and the triboelectric material even under very small deformation. Therefore, the BTUSE sensor maintains the process of electrostatic induction and contact electrification and thereby possesses a wider detection range. Additionally, according to the experiment on the location of the sensor, it can be found that sensors located closer to the muscles have stronger output signals (Note S12, Supporting Information). The comparative analysis of the performance between the BTUSE sensor and the existing HMI sensors is summarized in Table S1, Supporting Information. It can be seen that the BTUSE sensor has strong competitiveness among them, especially the characteristics of wider detection range and high sensitivity under a small trigger make it unique.

### 2.4 Applications of BTUSE Sensor in the Authentication System

**Figure 4:**
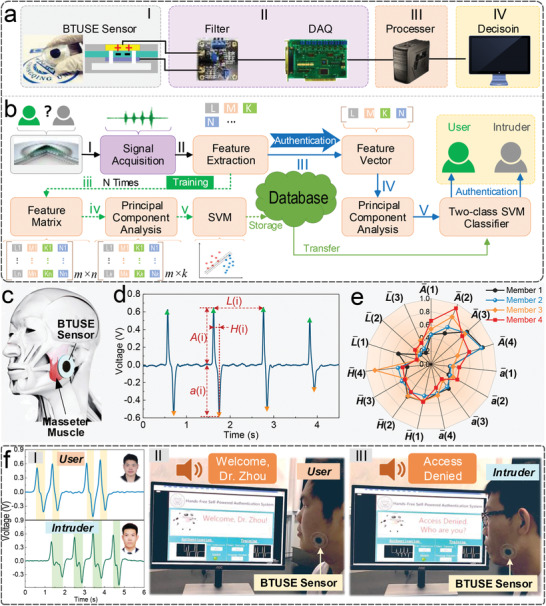
Application of BTUSE sensor in an authentication system. a) The hardware schematic of the system. b) The process flow of implementing authorization by using the proposed authentication system combined with the classification algorithm. c) Schematic diagram of BTUSE sensor mounting location. The training process: I, II, iii, iv, v. The authentication process: I–V. d) Signal of masseter muscle movement obtained by the BTUSE sensor to construct user profile models. Input latencies, holding time, and signal amplitudes are denoted as *L*, *H*, *A*, and *a*. e) Radar chart of four users’ normalized feature values after four movements of their masseter muscles. A total of 15 features, 3 input latencies, 4 holding time, and 8 signal amplitudes are extracted from the four triggers. f) Demonstration of the BTUSE sensor‐based authentication system. I) Different triggering habits of the user and the intruder. II) The user was successfully authorized by the system. III) The intruder was successfully denied access by the system.

The security of user privacy is the basic issue of communication. Before introducing the communication system, an intelligent authorization system based on the excellent performance of the BTUSE sensor above was first developed. In the system, the analog voltage signal generated by the sensor from muscle motion is converted into a digital signal through a hardware circuit composed of a filter and data acquisition system (DAQ) (**Figure** **4**a). Then, the signal information representing the muscle movement is analyzed and recognized in the software platform. The software platform is based on the support vector machine (SVM) algorithm and principal component analysis (PCA), and its workflow includes a training process and an actual authorization process (Figure 4b). During the training process (Figure 4b‐I,II,iii,iv,v), the acquired signals are combined into a matrix and the features are extracted through signal processing techniques such as denoising and peak detection. PCA is then used to extract the principal features and remove redundant information (Note S13, Supporting Information). Subsequently, a two‐class SVM classifier is applied to construct a user profile database for the principal features to support subsequent decision‐making. Adequate training of the classifier using the LIBSVM toolbox can improve the prediction accuracy. The trained classifier is then adopted for actual authorization. The authorization process is similar to the training process (Figure 4b‐I–V). The difference is that the function of the SVM classifier is converted to decide whether the feature of the collected signal matches the recorded data.

To demonstrate unrestricted mounting location, the BTUSE sensor is placed challengingly on the face with sharp skin curvature to sense the action of the masseter muscle (Figure 4c). The acquired signal has four main features, namely, input latencies (L), holding time (*H*), and signal amplitudes (*A*, *a*). A total of 15 features are collected after four triggers, and the radar chart of their normalized averages of four users indicates that different individuals have unique trigger behaviors (Figure 4d). To demonstrate the concept, two testers act as the user and the intruder respectively, and trigger four times as the input code for the recognition system (Figure 4e). The feature signals triggered by the user and the intruder are shown in Figure 4f‐I, and the user is fully trained to build user profiles for SVM before authorization. As shown in Video S2, Supporting Information, the user successfully obtains system authorization after four triggers (Figure 4f‐II), while the intruder who imitates the user to trigger four times is denied access (Figure 4f‐III). It means that an impostor cannot enter the system even if he knows the correct password, unless his trigger features match the information stored in the database. In short, with the assistance of machine learning, BTUSE sensors exhibit excellent security in protecting user privacy.

### 2.5 Applications of BTUSE Sensor in Personal Communication Aids

**Figure 5:**
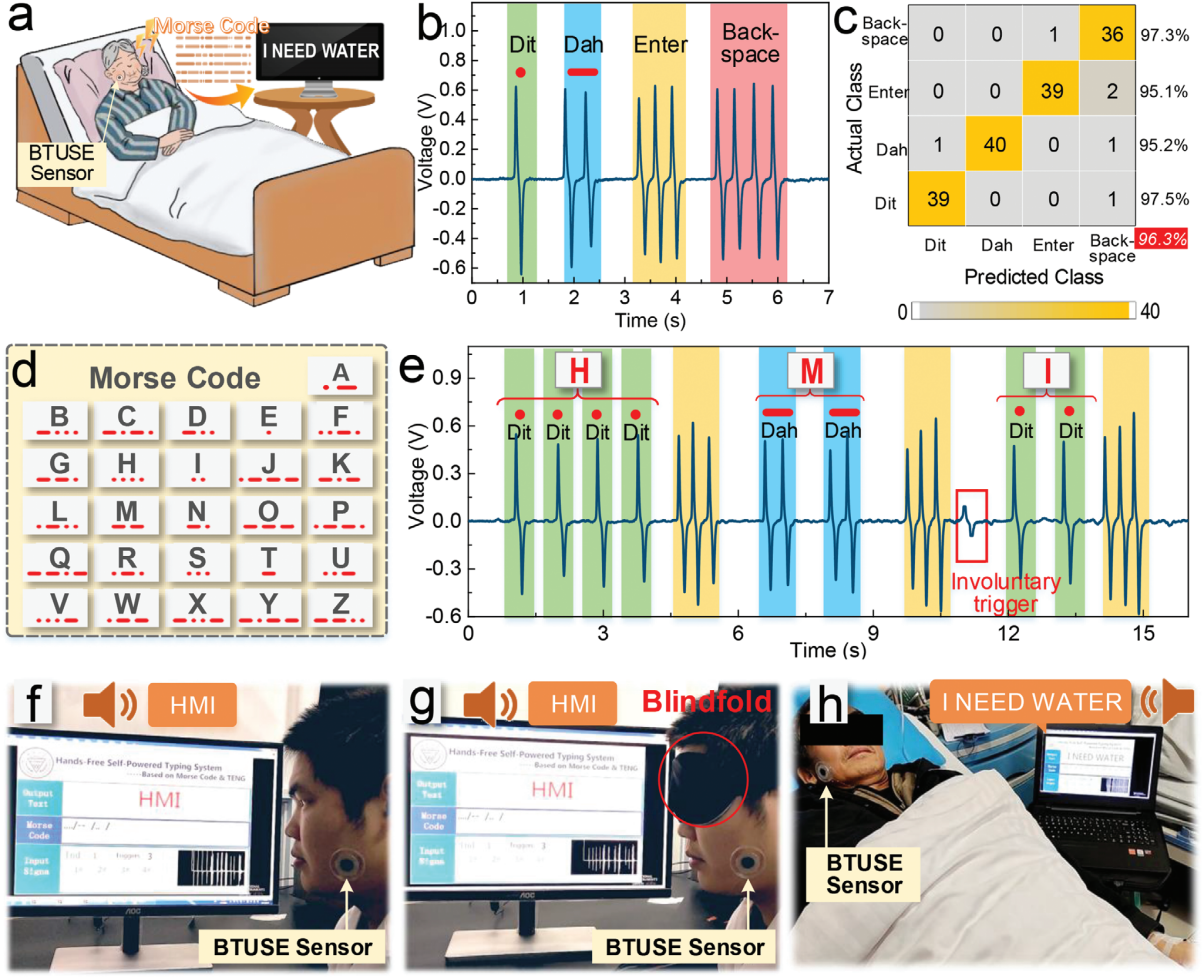
Application of BTUSE sensor in personal communication aids system. a) Sketch of the BTUSE sensor as a way to help the disabled communicate with the outside world, where the Morse code is chosen as the communication protocol. b) One trigger is set as the “dit” of Morse code, two triggers as the “dah”, three triggers as the “Enter”, and four triggers as the “backspace”. c) The confusion matrix for the four triggers. d) Morse code table of 26 English letters. e) Correspondence between the input signal and the word “HMI” in demonstrating a hands‐free personal communication aids system. Red dotted box: the involuntary trigger is ignored by adjusting the threshold of the trigger feature. f) Demonstration of the hands‐free personal communication aids system triggered by masseter muscle movement. g) Demonstration of blind input communication. h) Demonstration of helping patients communicate with the outside world in the hospital.

As an alternative to a traditional sEMG device, the BTUSE sensor has great potential in personal communication aids. Here, a hands‐free typing communication system based on the BTUSE sensor is developed to help the disabled communicate with the outside world (**Figure** **5**a). Considering that the output of the sensor is a single pulse signal, we use Morse code as the communication protocol for the first time to realize the HMI for the disabled. We set one trigger as “dit”, two triggers as “dah”, three triggers as “Enter”, and four triggers as “backspace” (Figure 5b). The time interval between “dit” and “dah” is a significant parameter. Short intervals require users to trigger quickly, while long intervals reduce the efficiency of communication. It means that each user has a unique optimal value due to different triggering habits. The optimal value is a compromise between efficiency and accuracy, and can be obtained by pre‐training with a machine learning algorithm. The high recognition accuracy of 96.3% is achieved after pre‐training in the SVM model, and the confusion matrix of the classification result is shown in Figure 5c. According to the general rules of Morse code, all letters can be represented by “dit” and “dah” (Figure 5d, Note S14, Supporting Information). In the demonstration, we successfully enter the word “HMI” according to the rules of Morse code (Figure 5e,f, Video S3, Supporting Information). It is effective to identify whether the user's trigger is voluntary or involuntary based on the value of the signal feature, and the involuntary trigger can be ignored by adjusting the threshold of the trigger feature (the red box in Figure 5e). As a result, the typing system exhibits high accuracy characteristics, and its confusion matrix showing the classification accuracy of the system is summarized in Table S2, Supporting Information. Another advantage of using Morse code as the communication protocol is that users can realize blind input communication as long as the Morse code table is remembered and confirm the input's correctness through the text‐to‐speech translation of the system (Figure 5g, Video S3, Supporting Information). It is friendly to the disabled, especially the blind, and provides an extremely competitive way for blind typing. To investigate its practicability, we found a patient in the hospital to communicate with the outside world through the system. The old man successfully entered the sentence “I NEED WATER” through the system to express his physiological needs to the outside world (Figure 5h, Video S4, Supporting Information). The prototype of the hands‐free typing system based on the BTUSE sensor demonstrated the potential for creating a new type of communication aid for medical and health care applications.

## 3 Conclusion

In conclusion, a bionic, ultra‐sensitive, sweat‐proof, and self‐powered sensor is reported for translating the real‐time micromotion of masseter muscle into a control command of HMI. The sensor is inspired by the croaking behavior of frogs and integrates self‐powered TENG technology, which successfully achieves the amplification of the wide micromotion of the masseter muscle to the significant motion of vibrating film, and finally generates high‐intensity electric signal output. The BTUSE sensor is unique in the working mechanism, which ingeniously integrates the self‐powered TENG technology and the bionic structure with an amplification effect. Compared with traditional sEMG electrodes, the BTUSE sensor has a higher signal‐to‐noise ratio and a more comfortable HMI operation. Compared with the traditional TENG sensor, the BTUSE sensor has a wider sensing range, since the contact‐separation action between the electrode and the triboelectric material always exists even under a small deformation. With the help of machine learning and using Morse code as the communication protocol, we construct two practical HMI systems based on the BTUSE sensor, namely an authorization system and a hands‐free typing system, to protect user privacy and help the disabled communicate with the outside world. This work provides a valuable toolkit for HMI applications of the disabled, and it brings new insights into the interdisciplinary cross‐integration between TENG technology and bionics.

## 4 Experimental Section

### Numerical Simulations

The structure movement and the potential distribution were numerically calculated using a commercial software COMSOL Multiphysics (5.3a version). To save simulation time and boost the modeling efficiency, a 2D axisymmetric model was used for simulation, and the modeled dimension was consistent with the actual device parameter. For the proposed BTUSE sensor, the authors' used the multiphysics model of fluid‐solid coupling (laminar flow and solid mechanics) for simulation. For the traditional TENG‐based two‐electrode sensor, the solid mechanic's module was employed for simulation. In the simulation of two‐electrode sensors, the boundary conditions of the contact pair were set to prevent mutual penetration of the films during the contact process. In addition, all potential distributions were simulated under the electrostatic field settings.

### Preparation of AgNWs/BaTiO_3_ NPs/PDMS Triboelectric Material

The preparation of AgNWs/BaTiO_3_ NPs/PDMS composite film is shown in Figure 2a. First, BaTiO_3_ NPs and AgNWs were dispersed in ethanol, then mixed into the PDMS prepolymer (Sylgard 184, Dow Corning), and stirred with a mechanical mixer for 20 min. After stirring, the curing agent was added to the mixed solution (the weight ratio of pre‐polymer and curing agent was 10:1), and then stirred with a mechanical mixer for 20 min to obtain a uniformly mixed suspension. Next, the mixed solution was poured into a rectangular groove mold, and an average thickness of 0.5 mm was achieved by the Doctor Blade technique. Finally, the mold containing the composite triboelectric material was placed in an oven at 60 °C for 3 h.

### Fabrication of the BTUSE Sensor

The TENG‐based BTUSE sensor consists of four parts: i) sensing film with PDMS support components; ii) AgNWs bottom electrode; iii) AgNWs/BaTiO_3_ NPs/PDMS friction layer; and iv) carbon‐based top electrode. The fabrication process of each part was as follows: i) the preparation of the sensing film began with the mixing of PDMS prepolymer and curing agent in a ratio of 10:1. Then, the well‐stirred PDMS was poured into a special mold and cured in an oven at 60 °C for 3 h. The mold was made of polymethyl methacrylate and cut into U‐shaped grooves by laser cutter (PLS6.75, Universal Laser Systems); ii) the preparation of AgNWs bottom electrode began with the forming of AgNWs film. First, AgNWs dispersion solution (Nanochem, China) was poured into a circular groove mold and cured in an oven at 100 °C for 30 min. Then, PDMS was added onto the surface of AgNWs film and the average thickness was controlled to 0.5 mm. Finally, the mold containing the film was placed in an oven at 60 °C for 3 h; iii) the preparation of AgNWs/BaTiO_3_ NPs/PDMS triboelectric material was as described above; iv) the preparation of carbon‐based top electrode began with the forming of the carbon‐based conductive film. First, carbon‐based electrically conductive ink (Jujo Chemical Co., Ltd., Japan) was added into a circular groove mold and cured in an oven at 120 °C for 30 min. After the curing, PDMS was added onto the surface of the carbon‐based conductive film and cured at 60 °C for 3 h. Notably, the thickness of all the films mentioned above was controlled by using the Doctor Blade technique. After preparing each part, they were assembled into a BTUSE sensor by using PDMS as the adhesive.

### Characterization and Measurement

The morphology and structure of the materials were characterized by using a field emission scanning electron microscopy (TESCAN MIRA3). The crystalline phases of BaTiO_3_ NPs and AgNWs were measured by an X‐ray diffractometer with Cu‐K*α* radiation, a wavelength of 1.5418 Å, and a scanning speed of 2° min^−1^ (PANalytical X'Pert, The Netherlands). A vibration platform and a displacement sensor (Meinaite, Germany) were used to control the deformation of the vibrating film for quantified measurement. The vibration platform consists of a vibration exciter (JZK‐40), a function signal generator (SINOLERA YE1311), and a power amplifier (YE5872A). The open‐circuit voltage, short‐circuit current, and transferred charge of BSNG were measured by a programmable electrometer (Keithley 6514) and oscilloscope (ROHDE & SCHWARZ RTO 2024). The data obtained from the electrometer was collected by the Data Acquisition Card (NI PCI‐6259) on a desktop computer. Besides, real‐time data acquisition control and analysis were performed using LabVIEW software. The performance characterization of AgNWs/BaTiO_3_ NPs/PDMS composite films was carried out by using a picking‐up vibration structure. The schematic diagram of the vibration measurement platform is shown in Note S15, Supporting Information. Except for the doping ratios (wt%), other experiment conditions (including film size, vibration frequency, vibration intensity, etc.) remain unchanged during the measurement.

### Signal Processing and Software Interface

The signal acquisition, digital filtering, and graphical user interface were all implemented using LabVIEW software (2016 version). The graphical user interface of the authentication system and the typing system is shown in Note S16, Supporting Information. There were two ways to reduce noise, including filter denoising and digital denoising. A two‐stage twin‐T 50‐Hz notch filter with tunable Q‐factor was adopted to eliminate power‐line noise signal and digital denoising was realized by using wavelet denoising algorithm in LabVIEW software. The feature extraction and PCA were implemented using MATLAB software (2016b version). The two‐class SVM classifier was built by using the LIBSVM toolbox. The details of the proposed algorithm for feature extraction and SVM‐based classification are displayed in Note S10, Supporting Information.

### Statistical Analysis

The normalization of the output signal of the sensor was performed by *x*(*i*)/*x*_max_, where *x*(*i*) and *x*_max_ represent the signal output and maximum value of the output, respectively. Statistics were performed using the software Origin (OriginLab, Northampton, USA).

### Study Participation

Prior to participation in the experiments, informed consent was obtained from the volunteer in all experiments.

## Conflict of Interest

The authors declare no conflict of interest.

## Supporting information

Supporting Information

Supplemental Video 1

Supplemental Video 2

Supplemental Video 3

Supplemental Video 4

## Data Availability

The data that support the findings of this study are available from the corresponding author upon reasonable request.
