# Supplementary material for: Bionic Ultra‐Sensitive Self‐Powered Electromechanical Sensor for Muscle‐Triggered Communication Application
Source: Adv Sci (Weinh). 2021 Jun 3;8(15):2101020. doi: 10.1002/advs.202101020 (PMC8336610; doi:10.1002/advs.202101020)
Supplement: Supplementary file 1 — Supporting Information [file ADVS-8-2101020-s002.pdf]

© 2021 Wiley-VCH GmbH

ADVANCED SCIENCE
Open Access

Supporting Information

for *Adv. Sci.*, DOI: 10.1002/advs.202101020

Bionic Ultra-Sensitive Self-Powered Electromechanical Sensor for Muscle-Triggered Communication Application

Hong Zhou, Dongxiao Li, Xianming He, Xindan Hui,Hengyu Guo*, Chenguo Hu, Xiaojing Mu*, Zhong Lin Wang*

Copyright WILEY-VCH Verlag GmbH & Co. KGaA, 69469 Weinheim, Germany, 2021.

Supporting Information

Bionic Ultra-Sensitive Self-Powered Electromechanical Sensor for Muscle-Triggered Communication Application

Hong Zhou, Dongxiao Li, Xianming He, Xindan Hui, Hengyu Guo*, Chenguo Hu, Xiaojing Mu*, Zhong Lin Wang*

Table of Contents

Note S1. Stress distribution of films with different shapes 3

Note S2. Velocity field in air and displacement of the BTUSE sensor 4

Note S3. Theoretical analysis of amplification effect in the BTUSE sensor 5

Note S4. Characterization of $BaTiO_3$ nanoparticles and AgNWs 9

Note S5. EDS Characterization of the AgNWs/BaTiO3 NPs/PDMS film 10

Note S6. Raw data on the performance of dielectric friction materials 11

Note S7. Simulation analysis of the performance of the BTUSE sensor 12

Note S8. Simulation verification of the influencing factors of the BTUSE sensor 13

Note S9. Frequency response of the BTUSE sensor 14

Note S10. Frequency response of the BTUSE sensor 15

Note S11. Simulation analysis of the performance of traditional TENG-based two-electrode sensor 16

Note S12. Simulation analysis of the performance of traditional TENG-based two-electrode sensor 17

Note S13. Algorithms used in training and authorization 18

Note S14. Correspondence between signals and 26 English letters 20

Note S15. Vibration measurement platform for characterizing composite films 22

Note S16. Software interfaces for different demonstrations 23

Table S1. Performance Comparison between proposed BTUSE sensor and previously reported sensors 24

Table S2. The confusion matrix showing the classification accuracy (%) for the hands-free typing test (Average accuracy: 93.9%) 25

Reference 26

Note S1. Stress distribution of films with different shapes

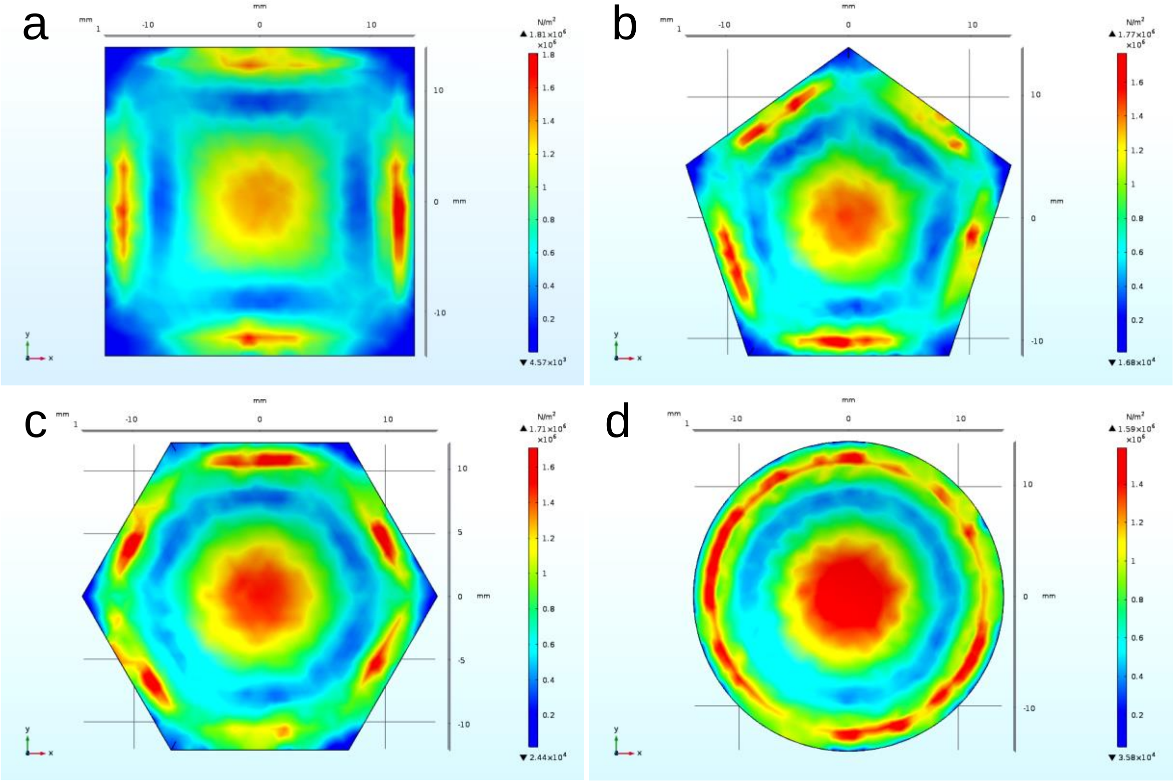

Maximum stress of different structures				
	Square structure	Pentagonal structure	Hexagonal structure	Circular structure
Maximum stress (N/mm^2)	1.81	1.77	1.71	1.59

Figure S1. The stress distribution of films with different shapes under the same force. a) Square structure. b) Pentagonal structure. c) Hexagonal structure. d) Circular structure. As observed, the film with a circular structure has the most uniform stress distribution and the smallest value.

Note S2. Velocity field in air and displacement of the BTUSE sensor

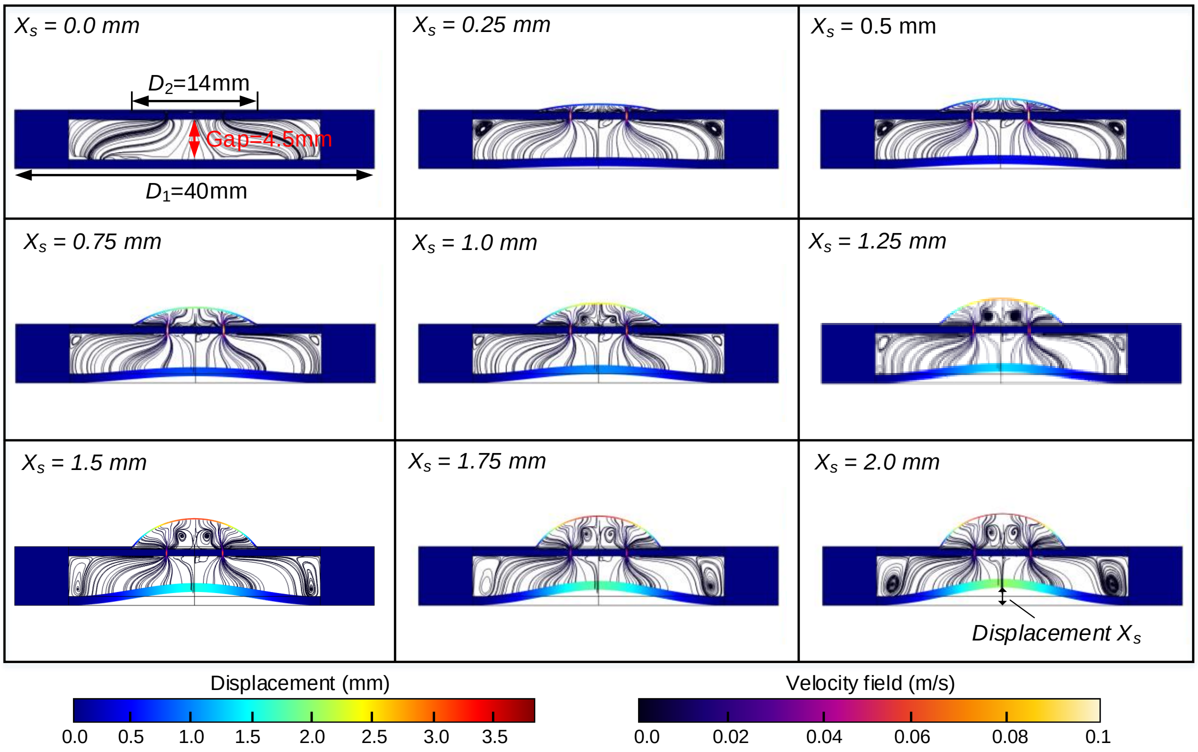

Figure S2. Velocity field in air and displacement of the vibrating film and the sensing film. The displacement difference between the vibrating film and the sensing film reveals the amplification effect in the BTUSE sensor.

Note S3. Theoretical analysis of amplification effect in the BTUSE sensor

During the working process of the BTUSE sensor, the vibrating film and sensing film move under the drive of muscles (**Supplementary Figure 3**a). Supplementary Figure 3b shows the corresponding vertical force diagram of the film. The force exerted by the muscle is simplified as a uniformly-distributed load due to the relatively small film area. It is essentially a problem of axisymmetric deformation of a circular film fixed on its edge under a uniformly-distributed load, which is also called *Hencky*'s problem[1]. The equilibrium condition of the film can be expressed as

$$2\pi r_s t_s \sigma_{sr} \sin\theta_s = \pi r_s^{\,2}\left(q - f_y\right) \tag{1}$$

where r_s is the radius of the point of the circular film we take, and t_s and σ_{sr} are film thickness and radial stress, respectively. f_{sy} is the vertical reaction force of air on the film when squeezed. Suppose the displacement of the film at r_s is $X_s(r)$, then

$$\sin\theta \cong -\frac{dX(r)}{dr} \tag{2}$$

Substituting Equation (2) into Equation (1), the equilibrium equation is obtained

$$\sigma_{sr} t_s \frac{dX_s(r_s)}{dr_s} = -\frac{r_s(q - f_{sy})}{2} \tag{3}$$

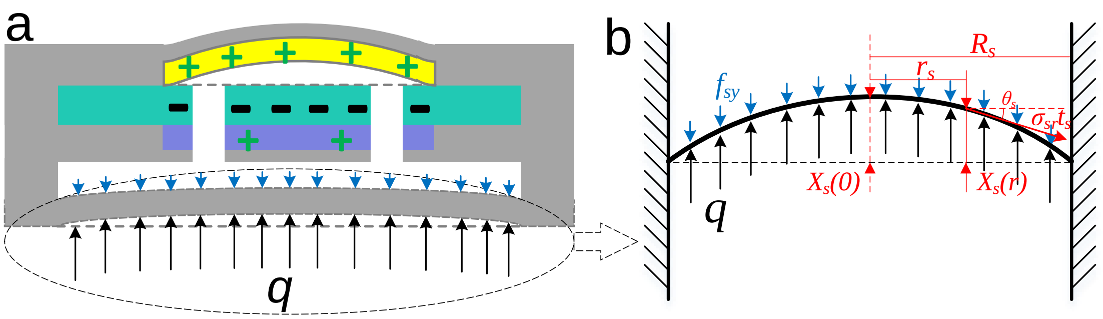

Figure S3. Film motion modeling of the BTUSE sensor. a) Schematic diagram of film motion of the BTUSE sensor. b) Corresponding vertical force diagram of the sensing film.

In the circular film plane, the equilibrium equation between the radial film force $\sigma_{st}t_s$ and the circumferential film force $\sigma_{st}t_s$ is

$$\frac{d(r_s t_s \sigma_{sr})}{dr_s} - t_s \sigma_{st} = 0 \tag{4}$$

The relationship between strain and displacement for large deflection problems is

$$\begin{cases} e_{sr} = \dfrac{du_s}{dr_s} + \dfrac{1}{2}\left(\dfrac{dX_s(r_s)}{dr_s}\right)^2 \\ e_{st} = \dfrac{u_s}{r_s} \\ \sigma_{sr} = \dfrac{E}{1-\upsilon^2}(e_{sr} + \upsilon e_{st}) \\ \sigma_{st} = \dfrac{E}{1-\upsilon^2}(e_{st} + \upsilon e_{sr}) \end{cases} \qquad \text{(5a, b, c, d)}$$

where e_{sr} and e_{st} are the radial strain and the circumferential strain, respectively, and u_s is the radial displacement. E and υ are Young's modulus of elasticity and Poisson's ratio, respectively. Solving the set of equations (4) and (5), we obtain the fundamental equations of large spatial deflection problems

$$\begin{cases} \dfrac{u_s}{r_s} = \dfrac{1}{Et_s}(t_s\sigma_{st} - \upsilon t_s \sigma_{sr}) = \dfrac{1}{Et_s}\left[\dfrac{d}{dr_s}(r_s t_s \sigma_{st}) - \upsilon t_s \sigma_{sr}\right] \\ r_s \dfrac{d}{dr_s}\left[\dfrac{1}{r_s}\dfrac{d}{dr_s}(r_s^2 t_s \sigma_{sr})\right] + \dfrac{Et_s}{2}\left(\dfrac{dX_s(r_s)}{dr_s}\right)^2 = 0 \end{cases} \qquad \text{(6a, b)}$$

By solving Equation (6), the transversal displacement $X_s(r_s)$ is obtained

$$X_s(r_s) = \left[\frac{R_s^4(q - f_{sy})\alpha}{2t_s E}\right]^{1/3}\left[g(\alpha) - g\left(\frac{\alpha r_s^2}{R_s^2}\right)\frac{r_s^2}{R_s^2}\right] \tag{7}$$

where $g(x)$ is a specific function,[1] and α is an undetermined integral constant. When $r_s = 0$, we obtain the maximum transversal displacement $X_s(0)$.

$$X_s(0) = \left[\frac{R_s^4\alpha\left(q - f_{sy}\right)}{2t_s E}\right]^{1/3} g(\alpha) \tag{8}$$

It can be seen from Equation (8) that the displacement is positively related to the radius R_s and inversely related to the thickness t_s.

Similar to the analysis of the sensing film, the maximum transversal displacement of the vibrating film can be expressed as (**Supplementary Figure 4**)

$$X_v(0) = \left[\frac{R_v{}^4 \beta f_{vy}}{2t_v E} \right]^{1/3} g(\beta) \tag{9}$$

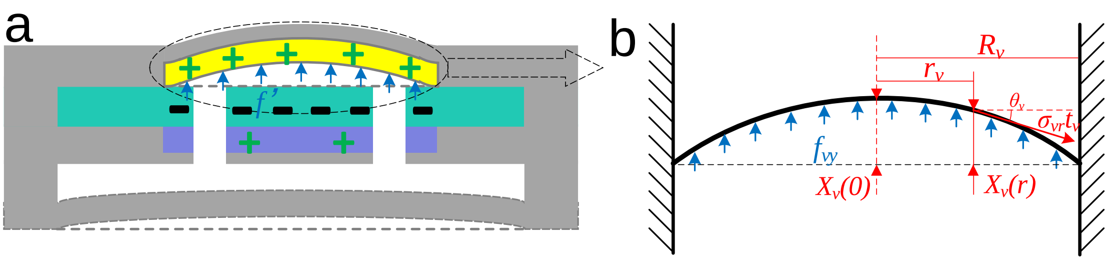

Figure S4. Film motion modeling of the BTUSE sensor. a) Schematic diagram of film motion of the BTUSE sensor. b) Corresponding vertical force diagram of the vibrating film.

Since the pressure of the vibrating film is the same as the pressure of the sensing film, we obtain

$$\frac{f_{sy}}{\pi R_s{}^2} = \frac{f_{vy}}{\pi R_v{}^2} \tag{10}$$

Then, solving the set of equations (8), (9), and (10), we obtain

$$\frac{2t_s E X_s(0)^3}{R_s{}^6 \alpha g(\alpha)} + \frac{2t_v E X_v(0)^3}{R_v{}^6 \beta g(\beta)} = \frac{q}{R_s{}^2 g(\alpha)} \tag{11}$$

$$\Gamma = \frac{X_v(0)}{X_s(0)} = \left(\frac{\beta}{\alpha}\right)^{\frac{1}{3}} \left(\frac{R_v}{R_s}\right)^2 \left[\frac{f_s}{q - f_s}\right]^{\frac{1}{3}} \frac{g(\beta)}{g(\alpha)} \tag{12}$$

Where $\Gamma = X_v(0)/X_s(0)$ represents the amplification effect in the BTUSE sensor. When the radius of the vibrating film is equal to that of the sensing film ($R_s = R_v$), there is no amplification effect in the BTUSE sensor ($X_s(0) = X_v(0)$). Therefore, we get $q/f_s = 1 +$

($\beta \cdot g(\beta)/\alpha \cdot g(\alpha)$). Clearly, Γ is related to $X_v(0)$ and $X_s(0)$. To observe the amplification effect's changing trend, we calculate the relationship between the magnification effect and the radius ratio when keeping q constant (**Supplementary Figure 5**). As observed, Γ is negatively correlated with the increase of $R_v(0)/R_s(0)$. That is, the amplification effect becomes more significant with the increasing dimension difference between the sensing film and the vibrating film.

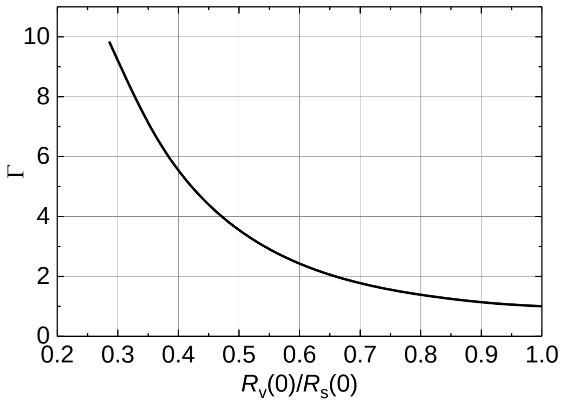

Figure S5. The relationship between the magnification effect and the radius ratio when keeping *q* constant.

Note S4. Characterization of $BaTiO_3$ nanoparticles and AgNWs

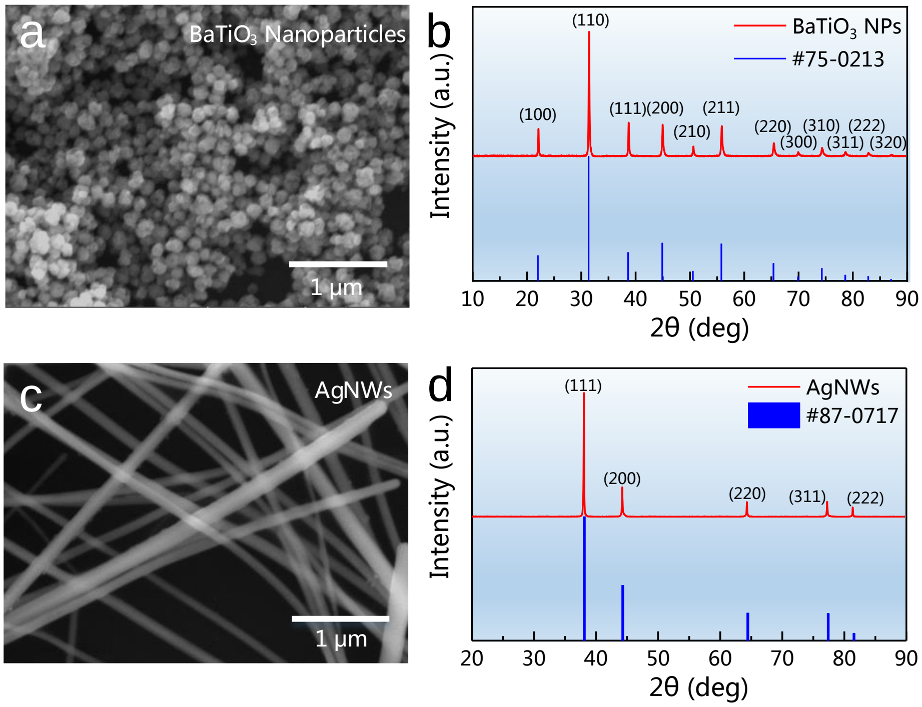

Figure S6. Characterization of $BaTiO_3$ nanoparticles (NPs) and AgNWs. a) SEM image of $BaTiO_3$ NPs. b) Measured XRD spectrum of $BaTiO_3$ NPs which agrees with the data reported in the card # 75-0213 of the powder diffraction file (PDF) database. c) SEM image of AgNWs. d) Measured XRD spectrum of AgNWs which agrees with the data reported in the card PDF # 87-0717. The consistency of the measured results with the database confirms the dopants.

Note S5. EDS Characterization of the AgNWs/BaTiO3 NPs/PDMS film

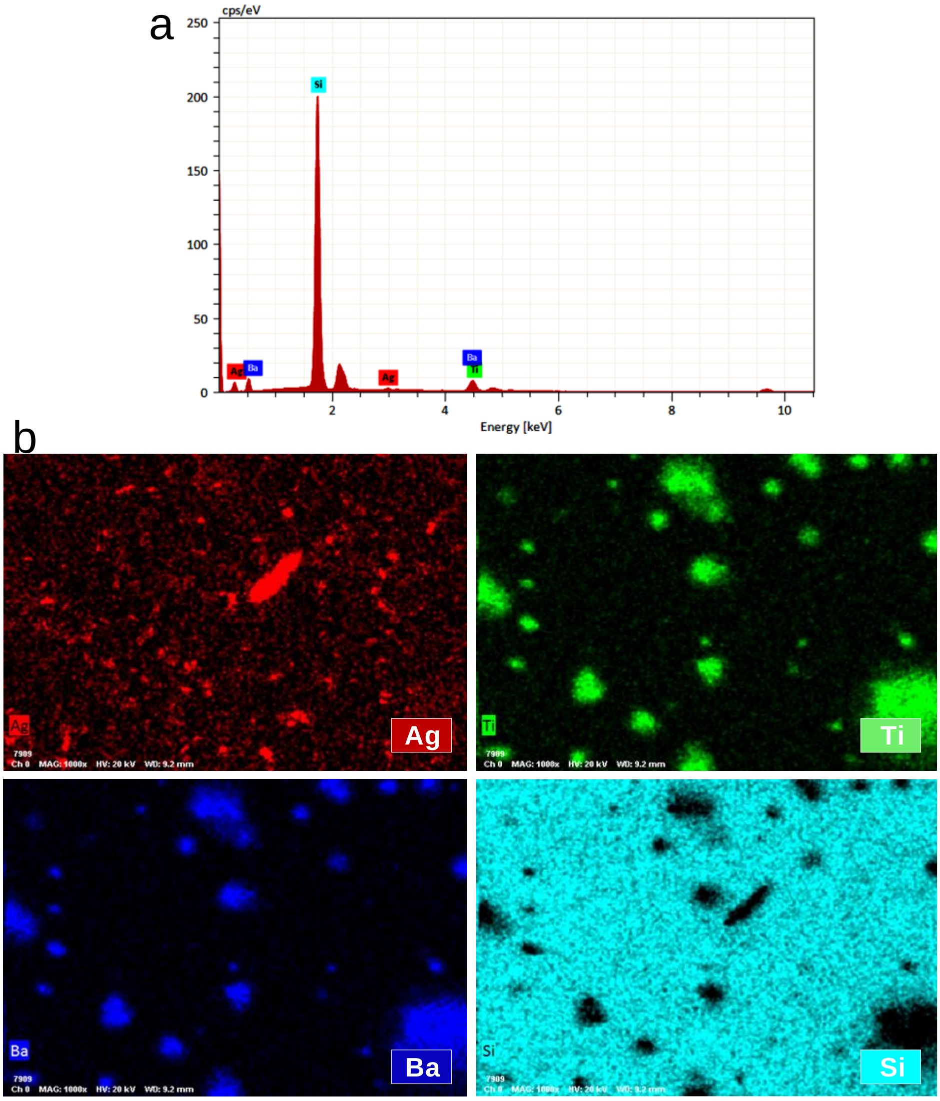

Figure S7. Energy dispersive X-ray spectroscopy (EDS) mapping of the cross-section of the AgNWs/$BaTiO_3$ NPs/PDMS composite film. a) Elemental composition and b) elemental mapping of the composite film. Ag elemental mapping represents the distribution of AgNWs in the composite film. Ti and Ba elemental mappings represent the distribution of BaTiO3 NPs in the composite film. Si elemental mapping represents the distribution of PDMS in the composite film.

Note S6. Raw data on the performance of dielectric friction materials

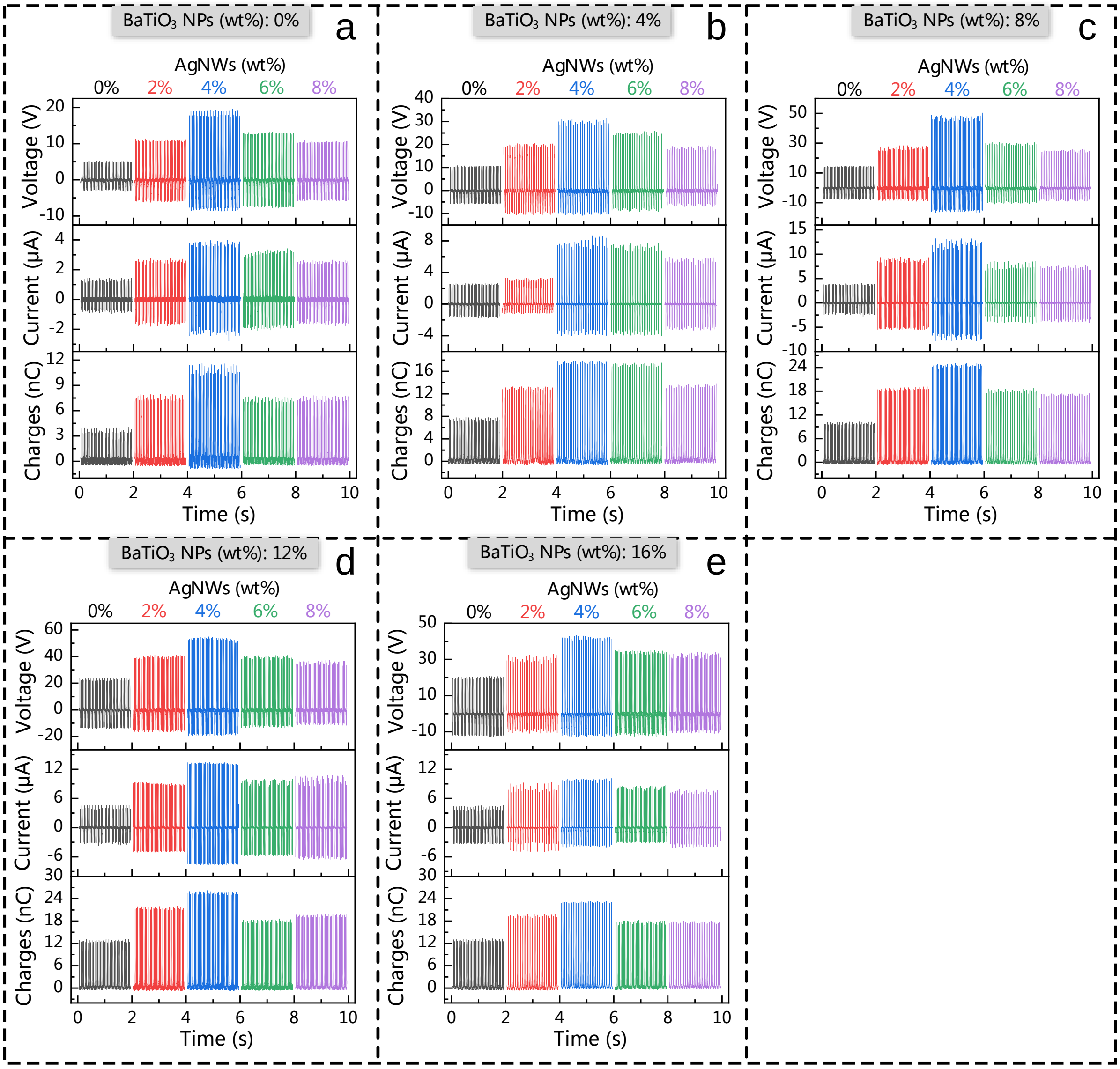

Figure S8. Raw data on the performance of dielectric triboelectric materials corresponding to the results in Figure 2 of the main text. Open-circuit voltage, short-circuit current, and transferred charge of AgNWs/$BaTiO_3$ NPs/PDMS composite film when the wt% of AgNWs ranges from 0% to 8% and that of $BaTiO_3$ NPs are a) 0%, b) 4%, c) 8%, d) 12%, e) 16%, respectively.

Note S7. Simulation analysis of the performance of the BTUSE sensor

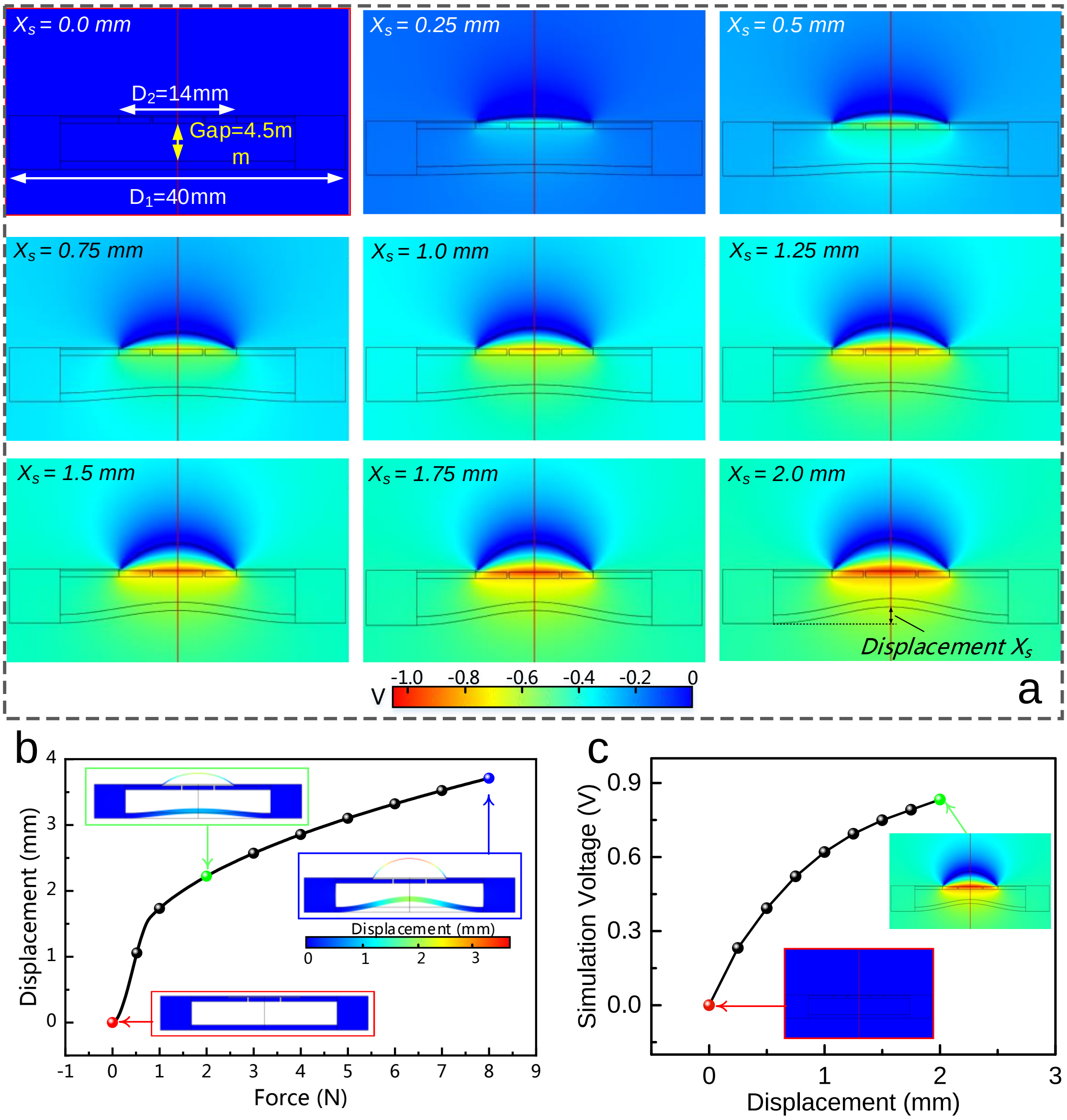

Figure S9. Simulation analysis of the performance of the BTUSE sensor. a) The voltage distribution on the sensor under different displacements. b) The displacement as a function of the force. c) The voltage as a function of the displacement. The red dot, green dot, and blue dot represent the output of the initial, intermediate, and saturated state, respectively.

Note S8. Simulation verification of the influencing factors of the BTUSE sensor

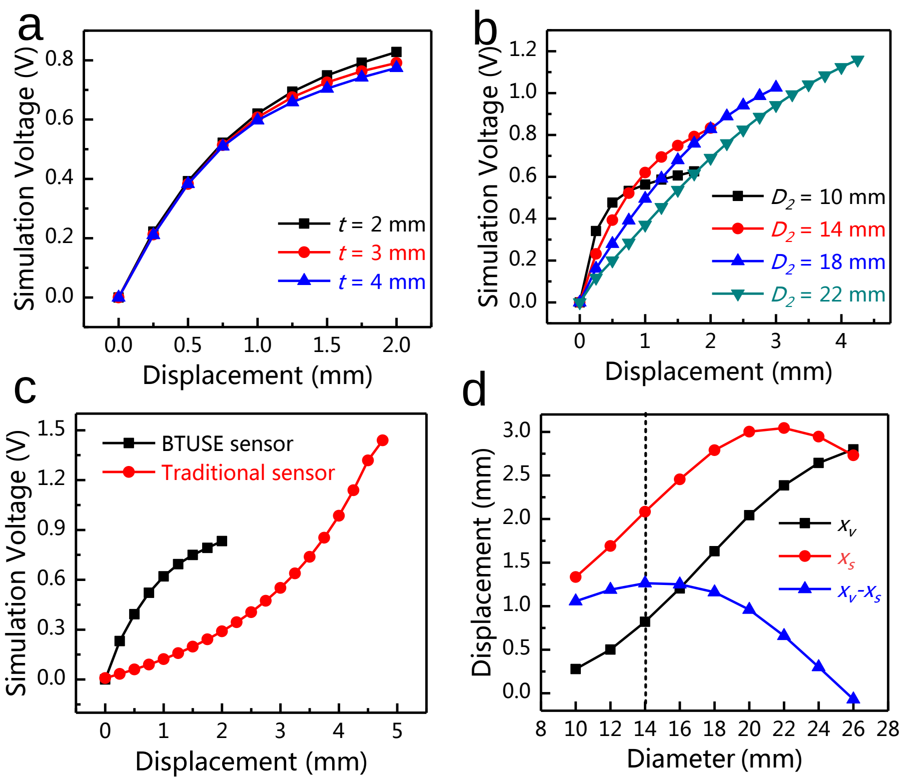

Figure S10. Simulation verification of the influencing factors of the BTUSE sensor. The influence of a) the film thickness and b) the film diameter on the performance. t and D_2 are the thickness and diameter of vibrating film, respectively. c) The performance comparison between the BTUSE sensor and the traditional TENG-based two-electrode sensor. d) The displacement as a function of the film diameter. X_s and X_v represent the displacement of the sensing film and the vibrating film, respectively. As observed, the BTUSE sensor with a film thickness of 0.2 mm, and a radius of 7 mm achieves high output and excellent sensitivity, which is consistent with the experimental results.

Note S9. Frequency response of the BTUSE sensor

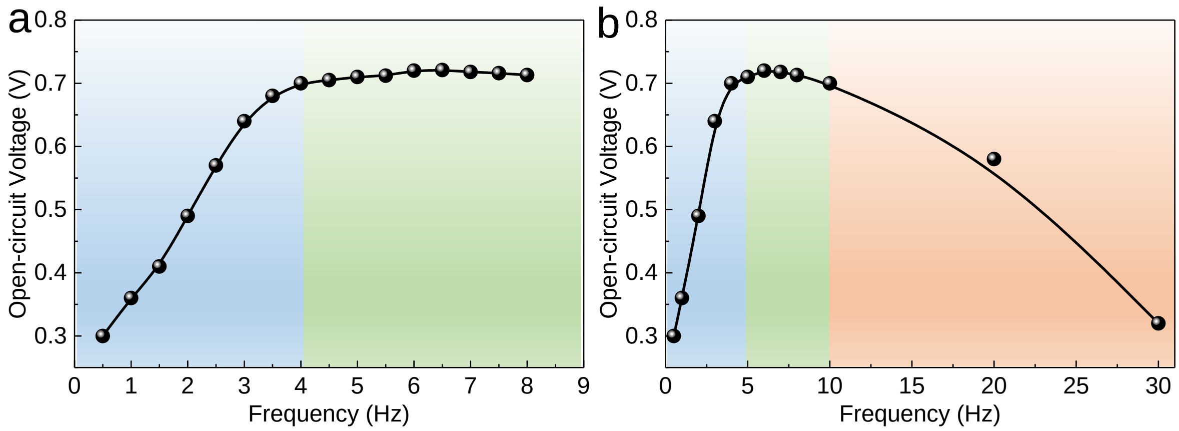

Figure S11. Frequency response of the BTUSE sensor. a) 0-8 Hz. b) 0-30 Hz. As observed, the output of the BTUSE sensor increases as the frequency rises before 4 Hz, and reaches saturation within 4-8 Hz. Beyond 8 Hz, the output of the sensor gradually decreases due to the influence of air damping.

Note S10. Frequency response of the BTUSE sensor

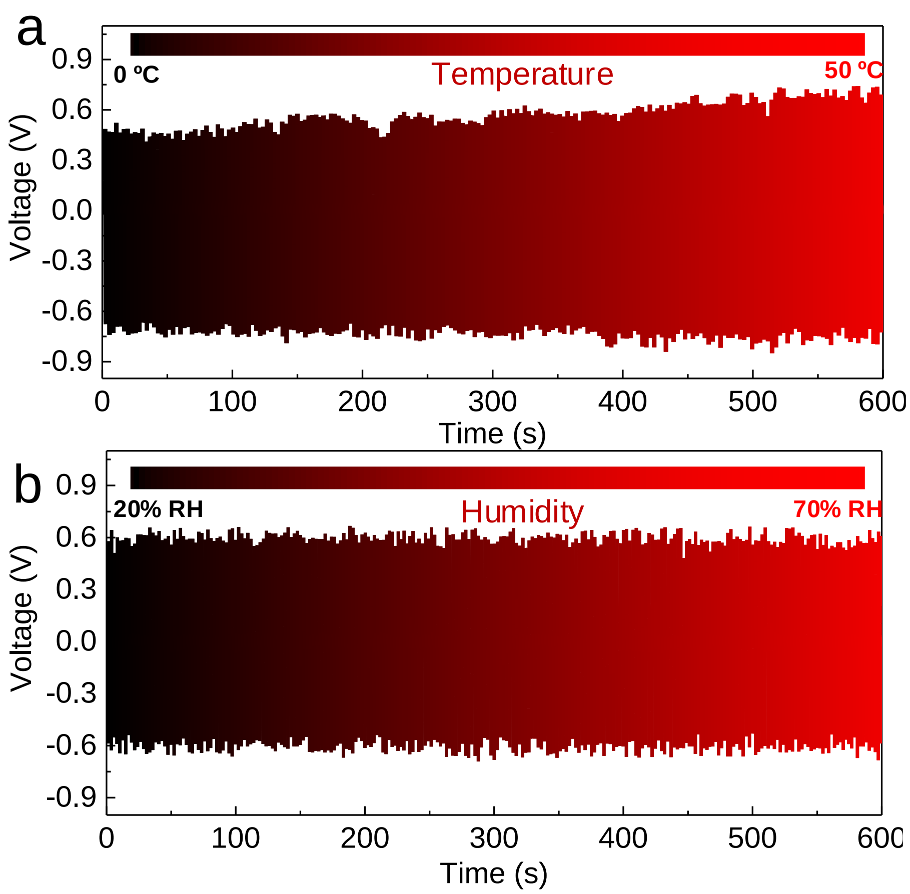

Figure S12. The influence of temperature and atmospheric humidity changes on the performance of the device. a) Temperature. b) Atmospheric humidity.

Figure S12a is the influence of temperature changes on the performance of the device. Clearly, the output voltage increases slightly as the temperature rises within the usual temperature range. This slight change will not affect the normal use of the sensor in the application system. Figure S12b is the influence of atmospheric humidity changes on the performance of the device. Clearly, no significant changes in the output voltage are observed when the humidity changes within the normal humidity range. This is because the entire device is covered and protected by the waterproof PDMS.

Note S11. Simulation analysis of the performance of traditional TENG-based two-electrode sensor

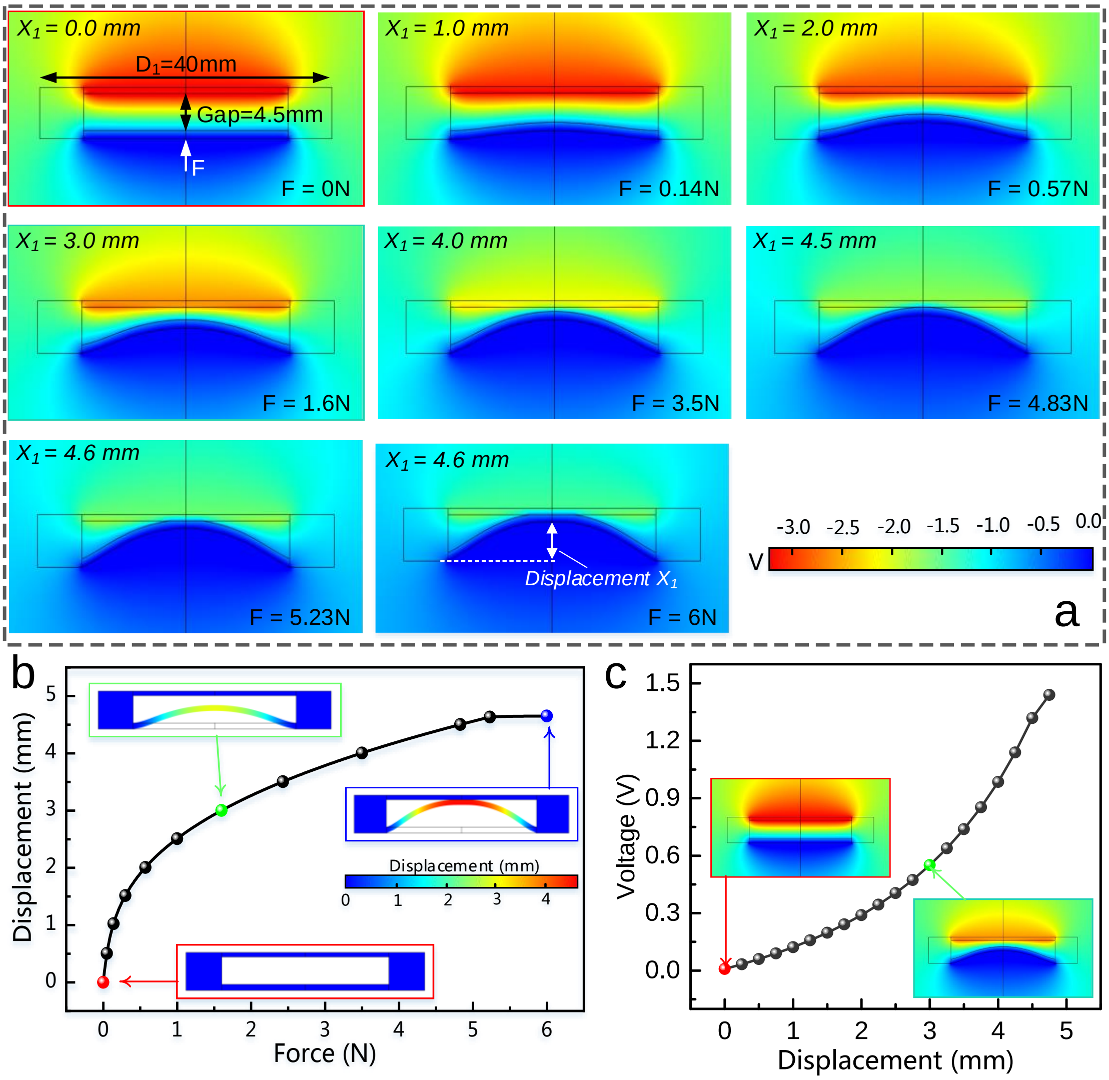

Figure S13. Simulation analysis of the performance of traditional TENG-based two-electrode sensor. a) The voltage distribution on the sensor under different displacements. b) The displacement as a function of the force. c) The voltage as a function of the displacement. The red dot, green dot, and blue dot represent the output of the initial, intermediate, and saturated state, respectively.

Note S12. Simulation analysis of the performance of traditional TENG-based two-electrode sensor

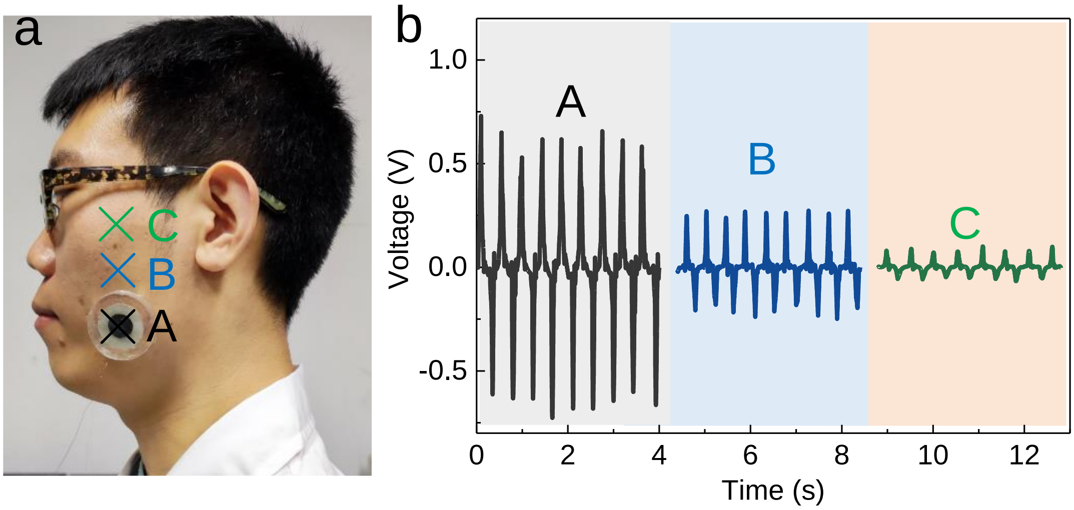

Figure S14. The influence of the fixed location of the sensor on the output signal. a) The location of the sensor on the face. b) Corresponding output signal.

Note S13. Algorithms used in training and authorization

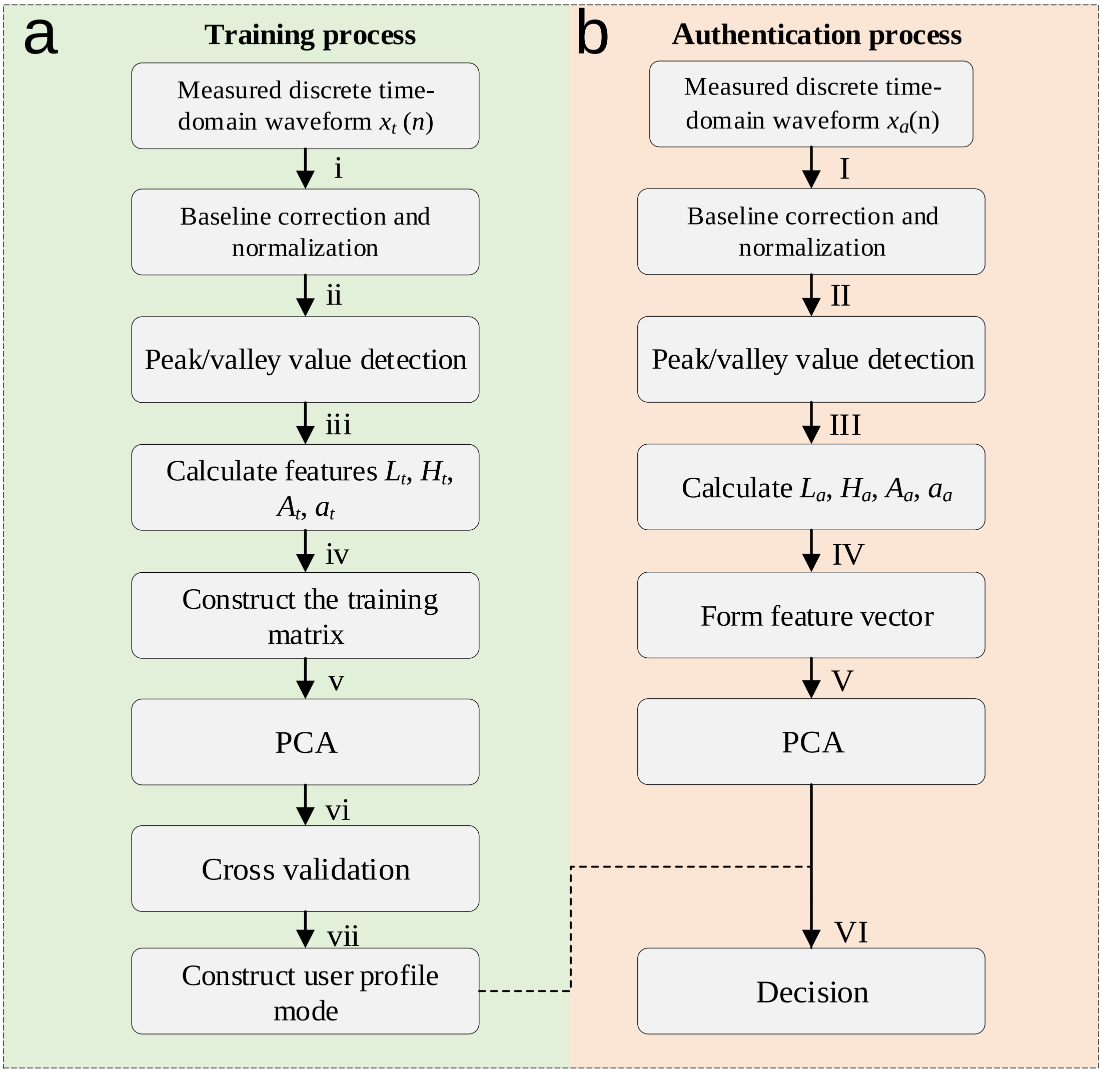

Figure S15. Feature extraction and two-class SVM classification construction. a) Training process. b) Authentication process.

1. Training process

i. $X_t(n) = \alpha \cdot [x_t(n) - b(n)]$, where $X_t(n)$, $x_t(n)$, $b(n)$, and α are calibrated and normalized waveform, initial waveform, baseline correction factor, normalization coefficient, respectively.

ii. $[X_{t\max}, i]$= $\max(X_t(n))$, where $X_{t\max}$ and i are the peak value and location index, respectively.

$[X_{t\min}, j]$= $\min(X_t(n))$, where $X_{t\min}$ and j are the valley value and location index, respectively.

iii. $L_t(i) = t_t(i+1) - t_t(i)$, where $t_t(i)$ represents the time point of the peak k.

$H_t(i) = X_{t\max}(i) – X_{t\min}(j)$. $A_t(i) = X_{t\max}(i)$. $a(i) = X_{t\min}(j)$.

iv. $\boldsymbol{M}$ = [$L_t(1)$, $L_t(2)$, $L_t(3)$, $H_t(1)$, $H_t(2)$, $H_t(3)$, $H_t(4)$, $A_t(1)$, $A_t(2)$, $A_t(3)$, $A_t(4)$, $a_t(1)$, $a_t(2)$, $a_t(3)$, $a_t(4)$]. After 50 times training, we obtain training matrix $\boldsymbol{P}_t = [\boldsymbol{M}_1, \boldsymbol{M}_2, \cdots, \boldsymbol{M}_{50}]^T$ and label vector $\boldsymbol{V}_t = [z_1, z_2, \cdots, z_{50}]$ (z_i = 0 or 1, where 0 and 1 represent rejection and acceptance, respectively.).

v. $\boldsymbol{N}_t$ = princomp($\boldsymbol{P}_t$), where princomp(x) function (PCA algorithm) is used to reduce the dimension of the feature matrix.

vi. The cross-validation is performed by importing $\boldsymbol{N}_t$ and $\boldsymbol{V}_t$ into LibSVM software[2].

vii. Obtain User Profile Model.

2. Authorization process

I. $X_a(n) = \alpha \cdot [x_a(n) - b(n)]$, where $X'(n)$, $x'(n)$, $b(n)$, and α are calibrated and normalized waveform, initial waveform, baseline correction factor, normalization coefficient, respectively.

II. [$X_{a\max}$, i]= max($X_a(n)$), where $X_{a\max}$ and i are the peak value and location index, respectively.

[$X_{a\min}$, j]= min($X_a(n)$), where $X_{a\min}$ and j are the valley value and location index, respectively.

III. $L_a(i) = t_a(i+1) – t_a(i)$, where $t_a(i)$ represents the time point of the peak k.

$H_a(i) = X_{a\max} (i) – X_{a\min} (j)$. $A_a(i) = X_{a\max} (i)$. $a_a(i) = X_{a\min} (j)$.

IV. $\boldsymbol{M}_a'$ = [$L_a(1)$, $L_a(2)$, $L_a(3)$, $H_a(1)$, $H_a(2)$, $H_a(3)$, $H_a(4)$, $A_a(1)$, $A_a(2)$, $A_a(3)$, $A_a(4)$, $a_a(1)$, $a_a(2)$, $a_a(3)$, $a_a(4)$].

V. $\boldsymbol{N}_a$ = princomp($\boldsymbol{M}_a$), where princomp(x) function (PCA algorithm) is used to reduce the dimension of the feature matrix.

VI. Decision by importing $\boldsymbol{N}_a$ and User Profile Model into LibSVM software[2].

Note S14. Correspondence between signals and 26 English letters

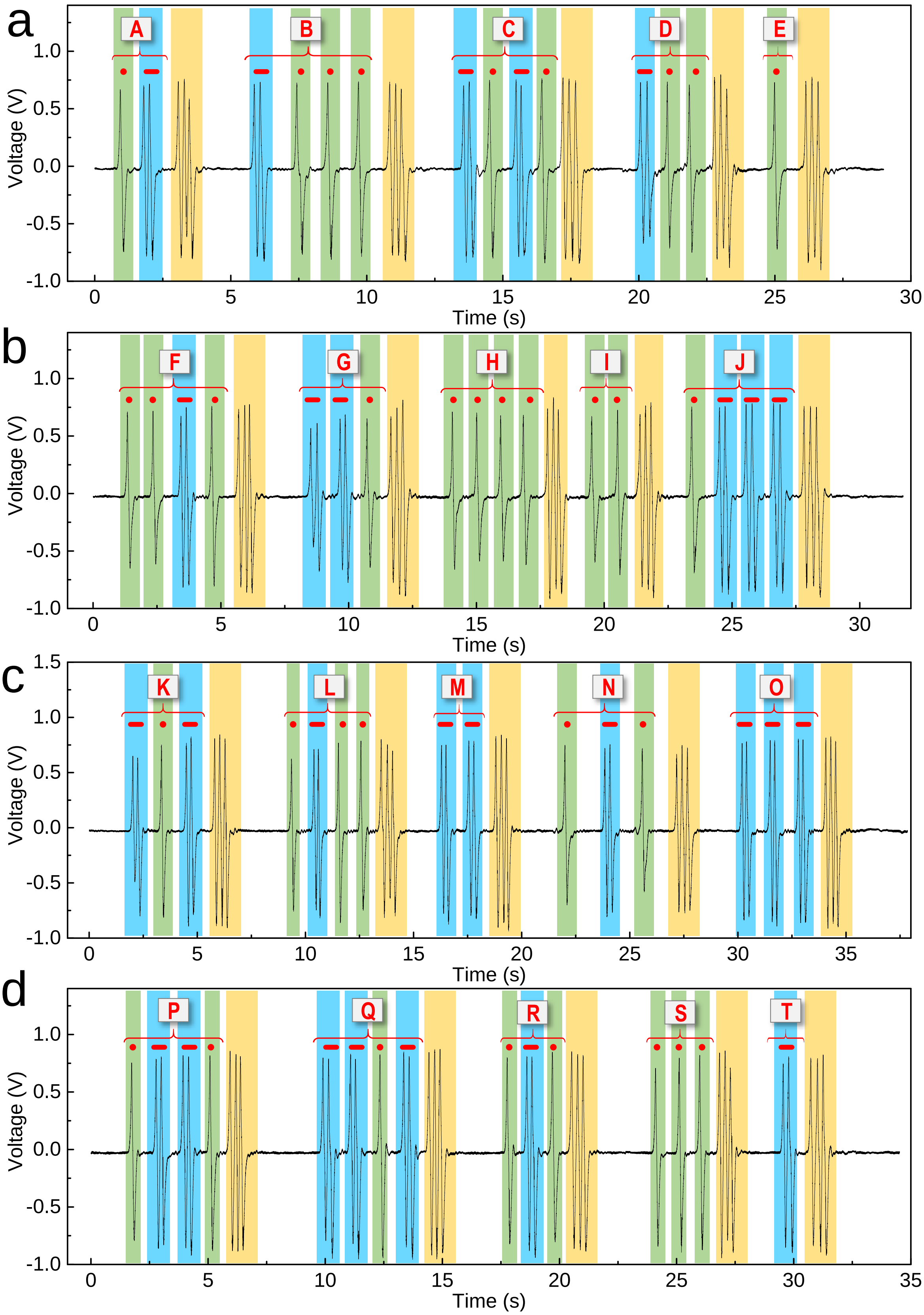

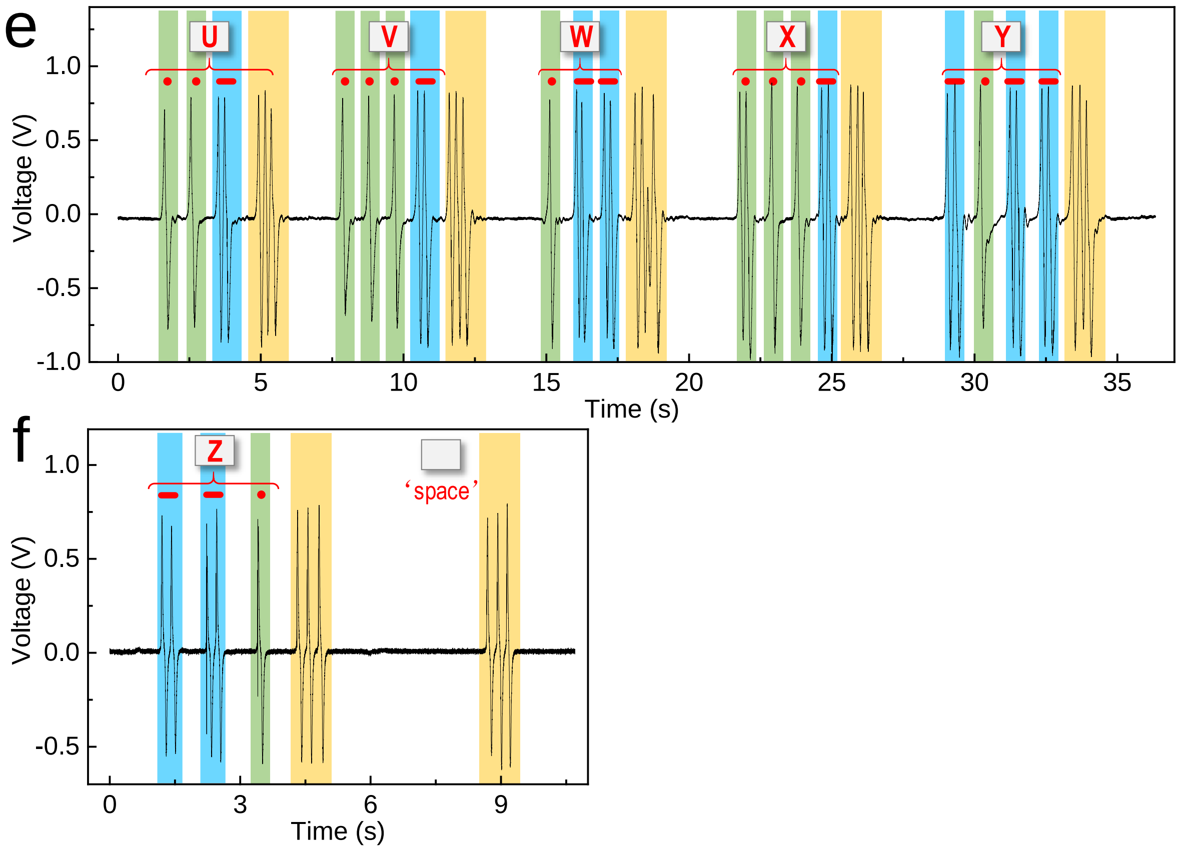

Figure S16. Correspondence between signals and 26 English letters. Each letter is entered by triggering three times (representing "Enter"). "Space" is entered by triggering "Enter" directly after the end of the last letter.

Note S15. Vibration measurement platform for characterizing composite films

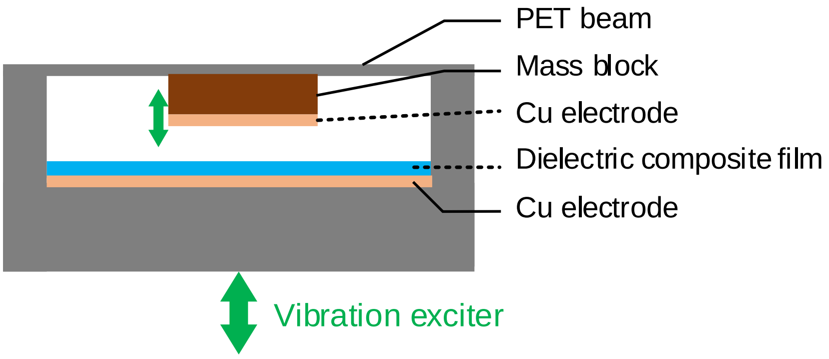

Figure S17. Schematic diagram of the vibration measurement platform for characterizing the performance of AgNWs/BaTiO3 NPs/PDMS composite films

Note S16. Software interfaces for different demonstrations

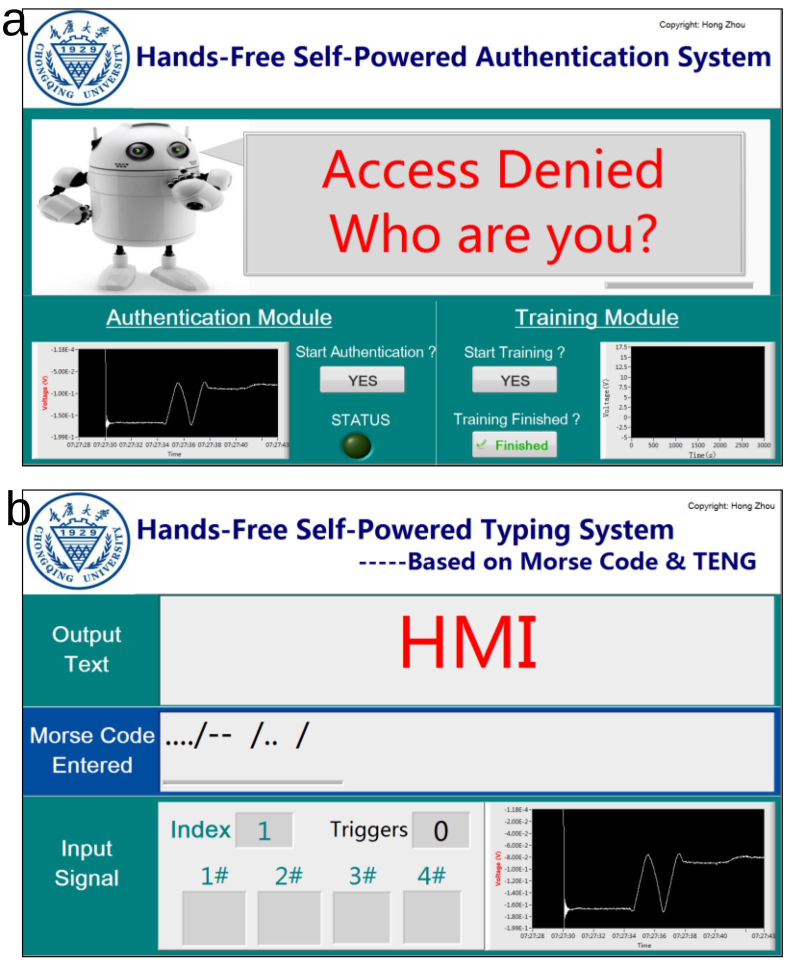

Figure S18. Software interfaces for different demonstrations. a) As an authentication system. b) As a typing system.

Table S1. Performance Comparison between proposed BTUSE sensor and previously reported sensors

References	Method	Flexibility	Performance	Self-powered	Remarks
Stepp *et al.* [3]	sEMG	×	Strength: 300 μV	×	Human-Machine Interface Control;<br>Uncomfortable user experience
Liu *et al.*[4]	Piezoresistive	√	Sensitivity:<br>~1.10 kPa^{-1}	×	Tactile sensor array;<br>High temperature dependency
Park *et al.*[5]	Capacitive	√	Sensitivity:<br>$\sim 1.78 \times 10^{-3}\ kPa^{-1}$	×	Pressure sensor array;<br>High resolution;
Rogers*et al.*[6]	Piezoelectric	√	Sensitivity:<br>~0.41-1.1 $VkPa^{-1}$	√	self-powered micro-mechanical elements;<br>Ultra-high sensitivity;
Hu *et al.*[7]	Triboelectric	√	Strength: ~750 mV	√	Eye motion triggered typing system;<br>Need a pair of glasses to fix the device;
This work	Triboelectric	√	Strength: ±700 mV;<br>Sensitivity: 54.6 mV/mm;<br>Sensing range: 0-5 mm;	√	Muscles triggered communication system;<br>Wider sensing range;<br>High sensitivity under a small trigger;<br>Suitable for blind people

Table S2. The confusion matrix showing the classification accuracy (%) for the hands-free typing test (Average accuracy: 93.9%)

Predicted class / Actual class	A	B	C	D	E	F	G	H	I	J	K	L	M	N	O	P	Q	R	S	T	U	V	W	X	Y	Z	Accuracy (%)
A	20	0	0	0	0	0	0	0	1	0	0	0	0	0	0	0	0	0	0	0	0	0	0	0	0	0	95.2%
B	0	19	0	0	0	0	0	0	0	0	0	0	0	0	0	0	0	0	0	0	0	0	0	2	0	0	90.5%
C	0	0	19	0	0	1	0	0	0	0	0	0	0	2	0	0	0	0	0	0	0	0	0	0	0	1	82.6%
D	0	0	0	19	0	0	0	0	0	0	0	0	0	0	0	0	0	0	0	0	0	0	0	0	0	1	95.0%
E	0	0	0	0	20	0	0	0	0	1	1	0	0	0	0	1	2	1	0	0	0	0	0	0	0	0	76.9%
F	0	0	0	0	0	19		0	0	0	0	0	0	0	0	0	0	0	0	0	0	1	0	0	0	0	95.0%
G	0	0	0	1	0	0	19	0	0	0	0	0	0	0	0	0	0	0	0	0	0	0	0	0	0	0	95.0%
H	0	0	0	0	0	0	0	18	0	0	0	0	0	0	0	0	0	0	0	0	0	0	0	0	0	0	100.0%
I	0	0	0	0	0	0	0	0	19	0	0	0	0	0	0	0	0	0	0	0	0	0	0	0	0	0	100.0%
J	0	0	0	0	0	0	0	0	0	18	0	0	0	0	0	0	0	1	0	0	0	0	0	0	0	0	94.7%
K	0	0	1	0	0	0	0	0	0	0	19	0	0	0	0	0	0	0	0	0	0	0	0	0	0	0	95.0%
L	0	0	0	0	0	0	0	0	0	0	0	19	0	0	0	0	0	0	0	0	0	0	0	0	0	0	100.0%
M	0	0	0	0	0	0	0	0	0	0	0	0	20	1	1	0	0	0	0	0	0	0	0	0	0	0	90.9%
N	0	0	0	0	0	0	0	0	0	0	0	0	0	17	0	0	0	0	0	0	0	0	0	0	1	0	94.4%
O	0	0	0	0	0	0	1	0	0	0	0	0	0	0	17	0	0	0	0	0	0	0	0	0	0	1	89.5%
P	0	0	0	0	0	0	0	0	0	0	0	1	0	0	0	18	0	0	0	0	0	0	0	0	0	0	94.7%
Q	0	0	0	0	0	0	0	0	0	0	0	0	0	0	0	0	18	0	0	0	0	0	0	0	2	0	90.0%
R	0	0	0	0	0	0	0	0	0	0	0	0	0	0	0	0	0	18	0	0	0	0	0	0	0	0	100.0%
S	0	0	0	0	0	0	0	0	0	0	0	0	0	0	0	0	0	0	20	0	0	0	0	1	0	0	95.2%
T	0	0	0	0	0	0	0	0	0	1	0	0	0	0	2	0	0	0	0	20	0	0	0	0	0	0	87.0%
U	0	0	0	0	0	0	0	0	0	0	0	0	0	0	0	0	0	0	0	0	20	0	0	0	0	0	100.0%
V	0	0	0	0	0	0	0	2	0	0	0	0	0	0	0	0	0	0	0	0	0	19	0	0	0	0	90.5%
W	0	0	0	0	0	0	0	0	0	0	0	0	0	0	0	1	0	0	0	0	0	0	20	0	0	0	95.2%
X	0	0	0	0	0	0	0	0	0	0	0	0	0	0	0	0	0	0	0	0	0	0	0	17	0	0	100.0%
Y	0	0	0	0	0	0	0	0	0	0	0	0	0	0	0	0	0	0	0	0	0	0	0	0	17	0	100.0%
Z	0	1	0	0	0	0	0	0	0	0	0	0	0	0	0	0	0	0	0	0	0	0	0	0	0	17	94.4%

Reference

[1] H. Hencky, Uber den Spannungszustand in kreisrunden Platten mit verschwindender Biegungssteifigkeit. *Zeitschrift fur Mathematik und Physik* **63**, 311-317 (1915).

[2] C. C. Chang, C. J. Lin, *ACM Trans. Intell. Syst. Technol.* **2011**, *2*, 1-27.

[3] J. M. Vojtech, G. J. Cler, C. E. Stepp, *IEEE Trans. Neural Syst. Rehabil. Eng.* **2018**, 26, 1566.

[4] L. Wang, H. Peng, X. Wang, X. Chen, C. Yang, B. Yang, J. Liu, *Microsyst. Nanoeng.* **2016**, 2, 16065.

[5] B. W. An, S. Heo, S. Ji, F. Bien, J.-U. Park, *Nat. Commun.* **2018**, 9, 2458.

[6] L. Persano, C. Dagdeviren, Y. Su, Y. Zhang, S. Girardo, D. Pisignano, Y. Huang, J. A. Rogers, *Nat. Commun.* **2013**, 4, 1633.

[7] X. J. Pu, H. Y. Guo, J. Chen, X. Wang, Y. Xi, C. G. Hu, Z. L. Wang, *Sci. Adv.* **2017**, 3, e1700694
